# Electronic Health Record Nudges and Health Care Quality and Outcomes in Primary Care: A Systematic Review

**DOI:** 10.1001/jamanetworkopen.2024.32760

**Published:** 2024-09-17

**Authors:** Oliver T. Nguyen, Avaneesh R. Kunta, Sri Varsha Katoju, Sara Gheytasvand, Niloofar Masoumi, Ronia Tavasolian, Amir Alishahi Tabriz, Young-Rock Hong, Karim Hanna, Randa Perkins, Arpan Parekh, Kea Turner

**Affiliations:** 1 Department of Health Outcomes and Behavior, H. Lee Moffitt Cancer Center & Research Institute, Tampa, Florida; 2 Department of Industrial and Systems Engineering, University of Wisconsin at Madison, Madison; 3 College of Medicine, University of Central Florida, Orlando; 4 Department of Community Health and Family Medicine, University of Florida, Gainesville; 5 Tabriz University of Medical Sciences, Tabriz, Iran; 6 College of Pharmacy, Tehran University of Medical Sciences, Tehran, Iran; 7 Department of Clinical Science and Nutrition, University of Chester, England; 8 Department of Oncologic Science, University of South Florida, Tampa; 9 Department of Gastrointestinal Oncology, H. Lee Moffitt Cancer Center & Research Institute, Tampa, Florida; 10 Department of Health Services Research, Management, and Policy, University of Florida, Gainesville; 11 Department of Family Medicine, University of South Florida, Tampa; 12 Department of Internal Medicine, H. Lee Moffitt Cancer Center & Research Institute, Tampa, Florida; 13 College of Medicine, University of Miami, Miami, Florida

## Abstract

**Importance:**

Nudges have been increasingly studied as a tool for facilitating behavior change and may represent a novel way to modify the electronic health record (EHR) to encourage evidence-based care.

**Objective:**

To evaluate the association between EHR nudges and health care outcomes in primary care settings and describe implementation facilitators and barriers.

**Evidence Review:**

On June 9, 2023, an electronic search was performed in PubMed, Embase, PsycINFO, CINAHL, and Web of Science for all articles about clinician-facing EHR nudges. After reviewing titles, abstracts, and full texts, the present review was restricted to articles that used a randomized clinical trial (RCT) design, focused on primary care settings, and evaluated the association between EHR nudges and health care quality and patient outcome measures. Two reviewers abstracted the following elements: country, targeted clinician types, medical conditions studied, length of evaluation period, study design, sample size, intervention conditions, nudge mechanisms, implementation facilitators and barriers encountered, and major findings. The findings were qualitatively reported by type of health care quality and patient outcome and type of primary care condition targeted. The Risk of Bias 2.0 tool was adapted to evaluate the studies based on RCT design (cluster, parallel, crossover). Studies were scored from 0 to 5 points, with higher scores indicating lower risk of bias.

**Findings:**

Fifty-four studies met the inclusion criteria. Overall, most studies (79.6%) were assessed to have a moderate risk of bias. Most or all descriptive (eg, documentation patterns) (30 of 38) or patient-centeredness measures (4 of 4) had positive associations with EHR nudges. As for other measures of health care quality and patient outcomes, few had positive associations between EHR nudges and patient safety (4 of 12), effectiveness (19 of 48), efficiency (0 of 4), patient-reported outcomes (0 of 3), patient adherence (1 of 2), or clinical outcome measures (1 of 7).

**Conclusions and Relevance:**

This systematic review found low- and moderate-quality evidence that suggested that EHR nudges were associated with improved descriptive measures (eg, documentation patterns). Meanwhile, it was unclear whether EHR nudges were associated with improvements in other areas of health care quality, such as effectiveness and patient safety outcomes. Future research is needed using longer evaluation periods, a broader range of primary care conditions, and in deimplementation contexts.

## Key Points

**Question:**

What is the association between electronic health record (EHR) nudges and health care quality and outcomes in primary care?

**Findings:**

In this systematic review including 54 randomized clinical trials, EHR nudges were associated with improvements in some areas of health care quality, such as descriptive and patient-centeredness measures. However, the evidence on other health care quality areas and clinical outcomes was less consistent.

**Meaning:**

These results suggest that EHR nudges may help improve clinicians’ documentation behaviors through higher completeness and accuracy of office notes.

## Teaser

This systematic review investigates whether there is an association between electronic health record (EHR) nudges and health care outcomes in primary care settings and describes implementation facilitators and barriers.

## Introduction

Primary care clinicians are pivotal in population health by lowering health care costs and reducing mortality globally.^1,2,3^ Despite its importance, primary care delivery varies substantially across settings. Across the many conditions treated in primary care, evidence-to-practice gaps exist.^4,5,6^ To address this, research has examined how to change clinician behavior to encourage the use of evidence-based care. Nudges, or interventions that subtly guide individuals toward specific behaviors while preserving freedom of choice, represent one approach of interest.^7^

Nudges have been increasingly studied for their potential use in facilitating behavior change among individuals.^8,9,10,11^ Nudges do not require extensive resources to implement and target automatic cognitive processing, which may be used in situations when time is limited (eg, primary care setting). Nudges may potentially affect primary care clinicians’ behaviors.^10,12^ For instance, studies have found that a variety of nudges (eg, public commitments, social norm feedback) can potentially improve antibiotic prescribing for viral infections.^10^ Consequently, these encouraging results have led some health systems to examine how to improve the reach and sustainability of nudge interventions.

Since adoption of electronic health record (EHR) systems is high,^13^ the EHR represents an additional medium to disseminate nudge interventions.^14^ The EHR also offers the opportunity to present the nudge to clinicians in their workflows and allows health systems to monitor the effectiveness of implemented nudges across different medical specialties and clinician types. Clinicians may also benefit from EHR nudges through the reduction of cognitive load, especially those that aim to provide reminders, offer preselected options at decision points for review, or provide additional contextual information (eg, relative costs of medications) at the point of decision-making. In turn, this may help, in part, facilitate desired clinician behaviors. In light of a growing number of randomized clinical trials (RCTs) evaluating EHR nudges in primary care, there is a growing need to summarize these findings and determine if the benefits of EHR nudges apply uniformly to the different types of conditions that primary care clinicians typically manage. To our knowledge, EHR nudges have only been reviewed in the inpatient setting,^11^ which may differ from those used in primary care settings due to differences in EHR workflows, patient complexity, and alert fatigue.

To address this research gap, we conducted a systematic review to summarize how clinician-facing EHR nudges are associated with health care quality (eg, documentation patterns, ordering behaviors) and patient outcomes in primary care settings. We also summarized implementation facilitators and barriers of EHR nudge interventions. Findings from this line of research may reveal future research areas and influence policies aimed at improving evidence-based care delivery in primary care.

## Methods

We reported this systematic review according to the Preferred Reporting Items for Systematic Reviews and Meta-analyses (PRISMA) reporting guideline.^15^ This review was registered with PROSPERO (CRD42022309599).

### Data Sources and Search

We considered clinician-facing EHR nudges to include subtle modifications integrated into an EHR’s choice architecture, specifically designed to influence medical decision-making without limiting clinicians’ autonomy. These nudges are intended to guide clinicians toward preferred actions or decisions (eg, generic formulations over brand-name drugs) while allowing them to disregard the suggestions if they are not suitable or practical for a given patient (eg, patient allergies, lack of insurance coverage). Clinician-facing EHR nudges could include order sets or default dosages that represent the health system’s preferred treatment pathways for a given clinical situation. They may include alerts and/or reminders designed to more easily complete a desired action (eg, adding immunization orders, adding diagnosis to EHR problem list) and redesigning checklists or dropdown menus in documentation templates to display the health system’s preferred options first. Accordingly, in consultation with a health sciences librarian, we developed a search strategy (eTable 1 in Supplement 1) that included search terms for clinicians, EHRs, and nudges.

On June 9, 2023, we ran the search strategy on MEDLINE, Embase, PsycINFO, CINAHL, and Web of Science to identify relevant peer-reviewed literature. Our inclusion criteria included articles written in English, conducted in primary care settings (ie, family medicine, general internal medicine, general pediatrics), used a RCT design, used nudges developed within the EHR environment, and evaluated how the nudge was associated with health care quality and patient outcome measures. We excluded articles that were nonempirical (ie, commentaries, other reviews) or did not explicitly discuss what clinicians could do if they disagreed with a recommendation. Lastly, we hand-searched the reference lists of included studies to identify other potentially relevant articles.

### Study Selection

Two reviewers were randomly assigned to independently screen the titles and abstracts of included studies. For full-text reviews, 2 reviewers were also randomly assigned to independently assess each article for eligibility. In both review stages, conflicts were resolved by a third reviewer. All reviews were conducted using the web-based software, Covidence.

### Data Extraction and Quality Assessment

For each included study, 2 reviewers abstracted the following elements: country, targeted clinician types, medical conditions studied, length of evaluation period, study design, sample size, intervention characteristics, nudge mechanisms, implementation facilitators and barriers encountered, and major findings. Since a nudging intervention could involve several mechanisms, we mapped identified nudges to the nudging ladder model used in a prior systematic review.^16^ We adapted the Risk of Bias 2.0 tools to evaluate the studies based on randomized clinical trial design (cluster, parallel, crossover). Our team scored studies from 0 to 5 points, where those that scored 0 to 1 point were considered high risk of bias, 2 to 3 points were considered moderate risk of bias, and 4 to 5 points were considered low risk of bias.

### Statistical Analysis

We were unable to conduct a meta-analysis because outcome definitions across studies differed immensely, multiple studies had missing information on precision estimates, and there was high prevalence of studies of moderate or high risk of bias.^17,18^ Thus, we used the narrative review approach to report results by outcome type and clinical concept.^17^ For health care quality, measure types were derived from a quality framework and included patient safety, effectiveness, patient-centeredness, timeliness, efficiency, and descriptive.^19^ For patient outcomes, measure types were formed after identifying themes in the findings and included patient-reported outcomes, patient adherence, and clinical outcomes. For clinical concept, we adapted a previously published taxonomy of the most common primary care conditions to classify disease types.^20^ For each reported finding, we included point estimates and *P* values or 95% CIs. We considered a 2-sided threshold of *P* < .05 as statistically significant. We also examined potential differences in findings if studies with high risk of bias were excluded from the analysis.

## Results

### Study Characteristics

Overall, we found 18 381 articles and included 54 studies in this systematic review. The Figure illustrates the outcomes of our review process. Most studies were located in the US (79.6% [43 of 54]) and had a cluster RCT study design (63.0% [34 of 54]). Over time, there was a general trend in an increasing number of studies that use a RCT design to evaluate the EHR nudge. Approximately 68.5% of studies (37 of 54) implemented the EHR nudge alongside another intervention (eg, providing education, providing reference or support materials). Of the 34 studies that reported which EHR vendor their nudge was implemented in, 15 (44.1%) used Epic. Most studies evaluated process measures with few studies attempting to assess clinical outcome measures. Furthermore, when examining primary care conditions targeted, most measures pertained to chronic care management (eg, diabetes, hypertension) and preventative care (eg, breast cancer screening). Although physicians were the most studied clinician type, 15 studies also examined other types of clinicians, including nurse practitioners, physician assistants, registered nurses, and medical assistants. Most studies (79.6% [43 of 54]) used EHR nudges to facilitate the uptake of a targeted behavior while the remaining studies (20.4% [11 of 54]) used EHR nudges to facilitate a decrease in a targeted behavior. The number of studies that reported an association with improved outcomes was higher among studies facilitating an increase in a targeted behavior compared with those facilitating a decrease in a targeted behavior (54.7% vs. 9.1%). Lastly, only 7 studies formally assessed implementation factors. All of these studies identified implementation barriers, but none reported implementation facilitators (eTable 2 in Supplement 1). Table 1 displays a summary of the proportion of studies that showed positive, negative, and null associations of EHR nudges and each outcome type. Detailed results on how EHR nudges were associated with health care quality and outcome measures are shown in Table 2 and Table 3, respectively.

**Figure.**
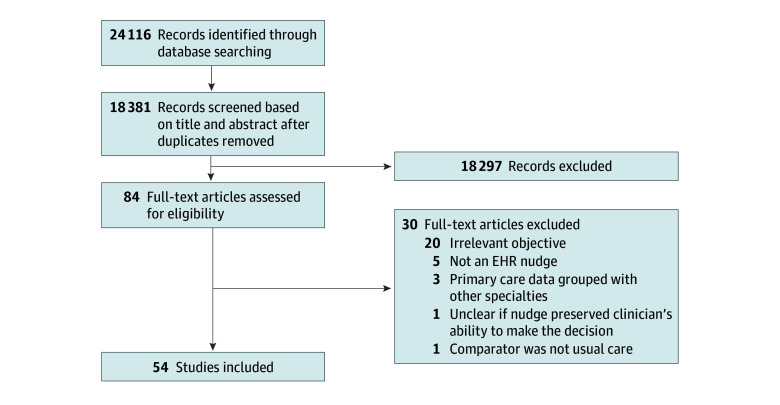
Study Flow Diagram EHR indicates electronic health record.

**Table 1.** Direction of Associations Between Electronic Health Record Nudges and Health Care Quality and Patient Outcomes

Outcome type	Total No. of measures	No. (%)			
		No. of measures with associations		No. of measures	
		With improved outcomes	With worsened outcomes	With mixed associations	With null associations
Patient safety	12	4 (33.3)	0	2 (16.7)	6 (50.0)
Effectiveness	48	19 (39.6)	0	13 (27.1)	16 (25.0)
Patient-centeredness	3	3 (100.0)	0	0	0
Timeliness	0	NA	NA	NA	NA
Efficiency	4	0	0	0	4 (100.0)
Descriptive	38	30 (78.9)	0	1 (2.6)	7 (18.4)
Patient-reported outcomes	3	0	0	1 (33.3)	2 (66.7)
Patient adherence	2	1 (50.0)	0	0	1 (50.0)
Clinical outcomes	7	1 (14.3)	0	0	6 (85.7)

Abbreviation: NA, not applicable.

**Table 2.** Overall Findings of Association Between Electronic Health Record Nudges and Health Care Quality Measures

Measure type and concept	Citation(s)	Measures	Overall findings^a^
**Patient safety**			
Appropriate prescribing	Campbell et al,^21^ 2021	Anticholinergic discontinuation	7.8% vs 8.2%; *P* = .65
Appropriate prescribing	Kraemer et al,^22^ 2022	Opioid prescriptions at index visit	OR = 0.74 (95% CI, 0.46-1.18)
Appropriate prescribing	Kraemer et al,^22^ 2022	Continued use of opioid prescriptions	OR = 1.08 (95% CI, 0.94-1.24)
Appropriate prescribing	Kraemer et al,^22^ 2022	Concurrent opioid and benzodiazepine use	OR = 1.10 (95% CI, 0.94-1.29)
Appropriate prescribing	Tamblyn et al,^23^ 2012	Psychotropic prescription	Immediate-acting benzodiazepines: mean difference = −0.008 (95% CI, −0.05 to 0.03); long-acting benzodiazepines: mean difference = −0.006 (95% CI, −0.001 to −0.000); antidepressants: mean difference = −0.011 (95% CI, −0.03 to 0.01); immediate potency opiates: mean difference = 0.001 (95% CI, −0.00 to 0.01); low potency opiates: mean difference = −0.004 (95% CI, −0.01 to 0.00); anticonvulsants: mean difference = 0.006 (95% CI, −0.00 to 0.01); antipsychotics: mean difference = 0.005 (95% CI, −0.00 to 0.01) No.of psychotropic medications prescribed: mean difference = −0.02 (95% CI, −0.09 to 0.05)
Appropriate prescribing	Gill et al,^24^,2011; Abdel-Kader et al,^25^ 2011	Discontinued NSAIDs	Gill et al: OR = 1.18 (95% CI, 0.99-1.40); Abdel-Kader et al: OR = 1.43 (95% CI, 0.32-6.33)
Appropriate prescribing	Tamblyn et al,^23^ 2012	Drug-related injuries	Mean difference: −0.17 (95% CI, −0.32 to −0.02)
Appropriate prescribing	Flottorp et al,^26^ 2002; Høye et al,^27^ 2013; Meeker et al,^28^ 2016; Gulliford et al,^29^ 2019	Ordering antibiotics for viral infections	Flottorp et al: −3.0% difference, *P* = .003 (pharyngitis); −0.4% difference, *P* = .64 (urinary tract infections); Høye et al: OR = 0.72 (95% CI, 0.60-0.86) (upper respiratory infections); Meeker et al: DiD: −5% (95% CI, −7.8 to 0.1) (suggested alternatives mechanism); DiD: −7% (95% CI, −9.1 to −2.9) (accountable justification mechanism); Gulliford et al: IRR = 0.88 (95% CI, 0.78-0.99 (upper respiratory infections)
Appropriate prescribing	Gill et al,^24^ 2011	Provided guideline-concordant care	OR = 1.19 (95% CI, 1.01-1.42)
Appropriate prescribing	Tamblyn et al,^30^ 2003	Inappropriate prescribing of new medications	RR = 0.82 (95% CI, 0.69-0.98)
Appropriate prescribing	Tamblyn et al,^30^ 2003	Inappropriate discontinuation of medications	RR = 1.06 (95% CI, 0.89-1.26)
Appropriate prescribing	Fortuna et al,^31^ 2009	Prescribing of heavily marketed hypnotics	RR = 0.74 (95% CI, 0.57-0.96)
**Effectiveness**			
Diabetes	Sequist et al,^32^ 2005	Eye exam screening	HR = 1.38 (95% CI, 0.81-2.32)
Diabetes	Sequist et al,^32^ 2005	Receiving recommended diabetes care	OR = 1.30 (95% CI, 1.01-1.67)
Diabetes, hypertension	Sequist et al,^32^ 2005; Abdel-Kader et al,^25^ 2011; Sequist et al,^33^ 2018; Tamblyn et al,^34^ 2018	Antihypertensive prescriptions	Sequist et al (2005): HR = 1.42 (95% CI, 0.94-2.14) (ACE inhibitors for patients with diabetes); HR = (95% CI, 0.72 - 1.63); (β-blockers for CAD); Abdel-Kader et al: OR = 0.84 (95% CI, 0.50-1.41) (ACE or ARB for CKD); Sequist et al (2018): 76% vs 79%, *P* = .17 (ACE or ARB for high-risk CKD); 64% vs 65%, *P* = .57 (ACE or ARB for low-risk CKD); Tamblyn et al: RR = 1.65 (95% CI, 1.17-2.33) (diuretics for newly diagnosed hypertension); RR = 0.61 (95% CI, 0.43-0.86) (other antihypertensives for newly diagnosed hypertension); RR = 1.10 (95% CI, 0.73-1.66) (prescribing 1 antihypertensive for newly diagnosed hypertension); RR = 0.91 (95% CI, 0.60-1.37) (prescribing 2 or more antihypertensives for newly diagnosed hypertension); RR = 1.09 (95% CI, 0.79-1.52) (diuretic for established hypertension); RR = 0.91 (95% CI, 0.66-1.27) (other antihypertensives for established hypertension); RR = 1.13 (95% CI, 0.90-1.42) (1 antihypertensive prescribed for established hypertension); RR = 0.88 (95% CI, 0.70-1.11) (2 or more antihypertensives prescribed for established hypertension)
Diabetes, other (CAD, hyperlipidemia)	Sequist et al,^32^ 2005; Gill et al,^35^ 2009; Adusumalli et al,^36^ 2023	Statin prescriptions	Sequist et al: HR = 1.10 (95% CI, 0.65-0.85 (only patients with diabetes); Sequist et al: HR = 1.51 (95% CI, 1.05-2.17) (only patients with CAD); Gill et al: OR = 0.05, *P* > .05 Adusumalli et al: 5.5% difference (95% CI, 3.2%-8.1%)
Immunizations	Frank et al,^37^ 2004	Tetanus immunizations	RR = 1.89 (95% CI, 1.59-2.25)
Immunizations	Frank et al,^37^ 2004; Loo et al,^38^ 2011	Pneumococcal immunizations	Frank et al: RR = 1.70 (95% CI, 1.10-2.62); Loo et al: OR = 2.01 (95% CI, 1.30-3.11)
Immunizations	Frank et al,^37^ 2004	Measles, mumps, and rubella immunizations	RR = 1.25 (95% CI, 0.82-1.93)
Immunizations	Frank et al,^37^ 2004; Fiks et al,^39^ 2009; Loo et al,^38^ 2011; Szilagyi et al,^40^ 2015	Influenza immunizations	Frank et al: RR = 0.96 (95% CI, 0.78-1.18); Fiks et al: OR = 1.22 (95% CI, 0.94-1.61); Loo et al: OR = 1.53 (95% CI, 1.23-1.91); Szilagyi et al: OR = 0.93 (95% CI, 0.69-1.25); OR = 0.89 (95% CI, 0.69-1.16) (reported data across 2 sites separately)
Immunizations	Fiks et al,^41^ 2013; Szilagyi et al,^40^ 2015	HPV immunizations	Fiks et al: HR = 1.5 (95% CI, 1.2 - 2.0 (dose 1); HR = 1.0 (95% CI, 0.8-1.1) (dose 2); HR = 1.1 (95% CI, 0.9-1.3) (dose 3); Szilagyi et al: OR = 0.92 (95% CI, 0.60-1.40; OR = 0.96 (95% CI, 0.59-1.56) (dose 1 at 2 clinics); OR = 1.01 (95% CI, 0.57-1.77); OR = 1.06 (95% CI, 0.68-1.66) (dose 2 at 2 clinics); OR = 0.93 (95% CI, 0.69-1.25); OR = 1.13 (95% CI, 0.68-1.88) (dose 3 at 2 clinics)
Immunizations	Stockwell et al,^42^ 2015; Szilagyi et al,^40^ 2015; Stephens et al,^43^ 2021	Pediatric immunizations	Stockwell et al: RR = 0.90 (95% CI, 0.83-0.98) (up-to-date immunizations); Szilagyi et al: OR = 1.44 (95% CI, 0.82-2.56); OR = 1.16 (95% CI, 0.68-1.99) (Tdap from 2 clinics); OR = 1.15 (95% CI, 0.64-2.05); OR = 1.08 (95% CI, 0.82-1.41) (MCV4 at 3 clinics); Stephens et al: 3.7% difference (95% CI, 1.8%-5.6%) (among young children); 3.2% difference (95% CI, 0.6%-6.9%) (among adolescents); 0.8% difference, 95% CI, −0.3% to 1.8%) (under-immunizations)
Immunizations	Fiks et al,^39^ 2009	Having up-to-date influenza immunization	3.4% difference (95% CI, −1.4 to 9.1)
Other (ADHD)	Co et al,^44^ 2010	Assessing ADHD care	OR = 2.2 (95% CI, 1.2-4.0)
Other (asthma)	Bell et al,^45^ 2010	Prescribing asthma controller	Urban practices: 6% difference, *P* = .006; suburban practices: 14% difference, *P* = .03
Other (atrial fibrillation)	Karlsson et al,^46^ 2018	Anticoagulant prescription	70.3% vs 70.0%, *P* = .01
Other (atrial fibrillation)	McKie et al,^47^ 2020	Guideline-concordant care for atrial fibrillation	OR = 0.94 (95% CI, 0.15 - 5.94)
Other (CAD)	Sequist et al,^32^ 2005; Sequist et al,^48^ 2012	Aspirin prescriptions	Sequist et al (2005): HR = 2.36 (95% CI, 1.37-4.07) (CAD); Sequist et al (2012): 20% vs 18%, *P* = .43
Other (CAD)	Sequist et al,^32^ 2005	Receiving recommended coronary artery disease care	OR = 1.25 (95% CI, 1.01-1.55)
Other (chest pain)	Sequist et al,^48^ 2012	Echocardiograms	51% vs 48%, *P* = .33
Other (domestic violence screening)	Feder et al,^49^ 2011	Referral to domestic violence agency	IRR = 22.1 (95% CI, 11.5-42.4)
Other (GERD, drug side effects)	Player et al,^50^ 2010; Gill et al,^24^ 2011	Prescribing gastroprotective medications	Player et al: OR = 1.11 (95% CI, 0.86-1.43) (newly diagnosed GERD); OR = 1.37 (95% CI, 1.12-1.68) (established patients with GERD); Gill et al: OR = 1.33 (95% CI, 1.01-1.74 (among patients receiving NSAIDs)
Other (heart failure)	McKie et al,^47^ 2020	Guideline-concordant care for heart failure	OR = 7.6 (95% CI, 1.2-47.5)
Other (hyperlipidemia)	McKie et al,^47^ 2020	Guideline-concordant care for hyperlipidemia	OR = 1.1 (95% CI, 0.6-1.8)
Other (kidney disease)	Abdel-Kader et al,^25^ 2011; Sequist et al,^33^ 2018	Proteinuria assessment	Abdel-Kader et al: OR = 1.73 (95% CI, 0.77-3.87); Sequist et al: 71% vs 70%, *P* = .35 (patients with high risk of CKD); 45% vs 21%, *P* < .001 (patients with low risk of CKD)
Other (kidney disease)	Sequist et al,^33^ 2018	eGFR test	Patients with high risk of CKD: 89% vs 89%, *P* = .90; patients with low-risk of CKD: 82% vs 80%, *P* = .20
Other (kidney disease)	Sequist et al,^33^ 2018	Hemoglobin test	Patients with high risk of CKD: 73% vs 73%, *P* = .63; patients with low risk of CKD: 61% vs 61%, *P* = .87
Other (kidney disease)	Sequist et al,^33^ 2018	Phosphorous lab test	Patients with high risk of CKD: 49% vs 38%, *P* < .001; patients with low risk of CKD: 23% vs 13%, *P* < .001
Other (kidney disease)	Sequist et al,^33^ 2018	25-OH vitamin D lab	Patients with high risk of CKD: 53% vs 45%, *P* = .002; patients with low risk of CKD: 31% vs 24%, *P* = .004
Other (kidney disease)	Sequist et al,^33^ 2018	Calcium lab	Patients with high risk of CKD: 75% vs 69%, *P* = .01; patients with low risk of CKD: 59% vs 54%, *P* = .11
Other (kidney disease)	Sequist et al,^33^ 2018	Parathyroid hormone lab	Patients with high risk of CKD: 49% vs 39%, *P* < .001; patients with low risk of CKD: 24% vs 14%, *P* < .001
Other (kidney disease)	Sequist et al,^33^ 2018	Annual nephrology visit	Patients with high risk of CKD: 45% vs 34%, *P* < .001 Patients with low risk of CKD: 17% vs 11%, *P* = .001
Other (lab monitoring)	Palen et al,^51^ 2006; Feldstein et al,^52^ 2006	Monitoring laboratory values	Palen et al: 56.6% vs 57.1%, *P* = .31; Feldstein et al: HR = 2.5 (95% CI, 1.8-3.5), *P* < .001
Other (osteoporosis)	Loo et al,^38^ 2011	Bone density scan	OR = 1.43 (95% CI, 0.94-2.17)
Other (substance use)	Linder et al,^66^ 2009	Smoking cessation prescriptions	2.0% vs 2.0%, *P* = .40
Other (substance use)	Linder et al,^66^ 2009	Smoking counseling referrals	4.5% vs 0.4%, *P* < .001
Other (substance use)	Lee et al,^54^ 2023	Alcohol treatment initiation	7.8% vs 6.2%, *P* = .04
Other (substance use)	Lee et al,^54^ 2023	Positive alcohol screen	18.0% vs 5.0%, *P* < .001
Other (substance use)	Lee et al,^54^ 2023	Assessment DSM-5 Alcohol Symptom Checklist	80.9% vs 4.1%, *P* < .001
Other (weight issues)	Schriefer et al,^55^ 2009	Weight loss prescriptions	0.5% vs 0.2%, *P* = .86
Other (weight issues)	Schriefer et al,^55^ 2009	Weight loss program referrals	1.1% vs 1.3%, *P* = 1.00
Other (weight issues)	Schriefer et al,^55^ 2009	Bariatric surgery referrals	0.8% vs 0.6%, *P* = 1.00
Other (weight issues)	Schriefer et al,^55^ 2009	Combination therapy for patients with obesity	11.9% vs 6.6%, *P* = .01
Routine health maintenance (cancer screening)	Frank et al,^37^ 2004	Cervical smear test	RR = 1.09, 95% CI 0.91-1.29
Routine health maintenance (cancer screening)	Hsu et al,^56^ 2013	HPV screening	40.9% vs 1.1%, *P* < .001
Routine health maintenance (hepatitis screening)	Hsu et al,^56^ 2013; Chak et al,^57^ 2018; Chak et al,^58^ 2020	Hepatitis B screening	Hsu et al: 34.1% vs 0.0%, *P* < .001; Chak et al (2018): OR = 3.13 (95% CI, 2.18-4.48); Chak et al (2020): OR = 3.23 (95% CI, 2.24-4.67)
Routine health maintenance (hepatitis screening)	Federman et al,^59^ 2017	Hepatitis C screening	OR = 8.99 (95% CI, 7.57-10.70)
Routine health maintenance (wellness screening)	Frank et al,^37^ 2004; Sequist et al,^32^ 2005; van Wyk et al,^60^ 2008; Gill et al,^35^ 2009; O’Connor et al,^61^ 2011; Sequist et al,^33^ 2018	Lipids screening	Frank et al: RR = 0.89 (95% CI, 0.73-1.09); Sequist et al (2005): HR = 1.41 (95% CI, 1.15-1.72) (among patients with diabetes); HR = 0.99 (95% CI, 0.75-1.29) (among patients with CAD); van Wyk et al: RR = 1.76 (95% CI, 1.41-2.20) (dyslipidemia screening); RR = 1.40 (95% CI, 1.15-1.70) (dyslipidemia treatment); Gill et al: OR = 15.00, *P* < .05 (for patients of high risk); OR = 1.47, *P* > .05 (for patients of moderate risk); OR = 0.97, *P* > .05 (for patients of low risk); O’Connor et al: 3.3% difference, *P* = .14 (among patients with diabetes); Sequist et al (2018): 82% vs 83%, *P* = .24 (for patients of high risk of CKD); 72% vs 70%, *P* = .19 (for patients of low risk of CKD)
Routine health maintenance (wellness screening)	Frank et al,^37^ 2004; Sequist et al,^32^ 2005; O’Connor et al,^61^ 2011; Zera et al,^62^ 2015; Weiner et al,^63^ 2020	Diabetes screening	Frank et al: RR = 0.98 (95% CI, 0.65-1.48); Sequist et al: HR = 1.14 (95% CI, 0.89-1.46) (among patients with diabetes); O’Connor et al: 4.1% difference, *P* = .045 (among patients with diabetes); Zera et al: OR = 1.04 (95% CI, 0.79-1.38); Weiner et al: 0.72 vs 0.74, *P* = .07 (A1C tests); 1.55 vs 1.63, *P* = .49 (No.of glucose tests); 1.20 vs 1.22, *P* = .63 (No. of creatinine tests)
Routine health maintenance (wellness screening)	McDowell et al,^64^ 1989; Frank et al,^37^ 2004; O’Connor et al,^61^ 2011; Kharbanda et al,^65^ 2018	Measuring blood pressure	McDowell et al: 30.7% vs 21.1%, *P* < .001; Frank et al: HR = 1.02 (95% CI, 0.90-1.16); O’Connor et al: 0.8% difference, *P* = .28 (patients with diabetes); Kharbanda et al: 14.3% vs 10.6%, *P* = .07 (within 30 d of index visit); 26.0% vs 23.4%, *P* = .46 (within 90 d of index visit)
**Patient-centeredness**			
Other (weight issues)	Schriefer et al,^55^ 2009	Diet counseling for patients with obesity	14.0% vs 7.3%, *P* = .002
Other (weight issues)	Schriefer et al,^55^ 2009	Exercise counseling for patients with obesity	12.1% vs 7.1%, *P* = .02
Hypertension	Kressin et al,^67^ 2016	Blood pressure counseling	β = 1.10, *P* = .01
**Timeliness**			
NA	None	NA	NA
**Efficiency**			
Upper respiratory infections, urinary tract infections	Flottorp et al,^26^ 2002	Ordering labs for viral infections	Urinary tract infections: −5.1% difference, *P* = .05; pharyngitis: −0.5% difference, *P* = .64
Other (lab monitoring)	Lo et al,^69^ 2009	Appropriate ordering of labs	OR = 1.05 (95% CI, 0.75-1.46)
Other (chest pain)	Sequist et al,^48^ 2012	Cardiac stress testing	10% vs 9%, *P* = .40
Other (musculoskeletal pain)	Zafar et al,^68^ 2019	Lumbar spine MRI	Same-day orders: OR = 0.92 (95% CI, 0.68-1.25); orders within 30 d of visit: OR = 0.94 (95% CI, 0.70-1.25)
**Descriptive**			
Routine health maintenance (wellness screening)	Frank et al,^37^ 2004	Documenting allergies	RR = 1.81 (95% CI, 1.63-2.02)
Routine health maintenance (wellness screening)	Frank et al,^37^ 2004	Documenting weight	RR = 1.28 (95% CI, 1.13-1.44)
Routine health maintenance (wellness screening)	Frank et al,^37^ 2004	Documenting smoking status	RR = 1.12 (95% CI, 0.90-1.39)
Other (weight issues)	Schriefer et al,^55^ 2009; Tang et al,^70^ 2012	Diagnosis of obesity	Schriefer et al: 16.6% vs 10.7%, *P* = .02; Tang et al: OR = 4.1 (95% CI, 1.3-12.7)
Other (ADHD)	Co et al,^44^ 2010	Documenting ADHD symptoms	100% vs 61.3%, *P* < .001
Other (ADHD)	Co et al,^44^ 2010	Documenting ADHD treatment effectiveness	96.6% vs 54.8%, *P* < .001
Other (ADHD)	Co et al,^44^ 2010	Documenting ADHD adverse events	96.6 vs 40.3%, *P* < .001
Other (GERD)	Player et al,^50^ 2010	Diagnosis of GERD	OR = 1.33 (95% CI, 1.13-1.56)
Other (asthma)	Bell et al,^45^ 2010	Filing up-to-date asthma care plan	Urban practices: 1% difference, *P* > .05; suburban practices: 25% difference, *P* = .03
Other (asthma)	Bell et al,^45^ 2010	Documenting spirometry	Urban practices: 3% difference, *P* = .04; suburban practices: 13% difference, *P* = .003
Other (kidney disease)	Abdel-Kader et al,^25^ 2011	CKD documentation	OR = 1.23 (95% CI, 0.60-2.51)
Other (domestic violence screening)	Feder et al,^49^ 2011	Documenting domestic violence	IRR = 3.1 (95% CI, 2.2-4.3)
Other (weight issues)	Tang et al,^70^ 2012	Documenting results of weight counseling	OR = 2.1 (95% CI, 1.1-4.1)
Other (ADHD)	Wright et al,^71^ 2012	ADHD problem list	OR = 2.23 (*P* < .0001)
Other (asthma)	Wright et al,^71^ 2012	Asthma/COPD problem list	OR = 2.98 (*P* < .0001)
Other (cancer)	Wright et al,^71^ 2012	Breast cancer problem list	OR = 1.78 (*P* < .001)
Other (CAD)	Wright et al,^71^ 2012	CAD problem list	OR = 4.66 (*P* < .0001)
Other (coagulopathy)	Wright et al,^71^ 2012	Congenital coagulopathy problem list	OR = 2.06, *P* = .04, finding not significant due to a Bonferroni correction
Other (CHF)	Wright et al,^71^ 2012	CHF problem list	OR = 7.56, *P* < .0001
Diabetes	Wright et al,^71^ 2012	Diabetes mellitus problem list	OR = 1.97, *P* < .0001
Other (glaucoma)	Wright et al,^71^ 2012	Glaucoma problem list	OR = 3.78, *P* < .0001
Hypertension	Wright et al,^71^ 2012	Hypertension problem list	OR = 4.12, *P* < .0001
Other (thyroid issues)	Wright et al,^71^ 2012	Hyperthyroidism problem list	OR = 1.30, *P* = .29
Other (thyroid issues)	Wright et al,^71^ 2012	Hypothyroidism problem list	OR = 3.99, *P* < .0001
Other (autoimmune issues)	Wright et al,^71^ 2012	Myasthenia gravis problem list	OR = 2.10, *P* = .11
Other (osteoporosis)	Wright et al,^71^ 2012	Osteoporosis/Osteopenia problem list	OR = 3.40, *P* < .0001
Other (autoimmune issues)	Wright et al,^71^ 2012	Rheumatoid arthritis problem list	OR = 3.97, *P* < .0001
Other (kidney issues)	Wright et al,^71^ 2012	Renal failure/insufficiency problem list	OR = 8.22, *P* < .0001
Other (sickle cell disease)	Wright et al,^71^ 2012	Sickle cell disease problem list	OR = 1.66, *P* = .29
Other (stroke)	Wright et al,^71^ 2012	Stroke problem list	OR = 2.35, *P* = .0002
Other (social determinants of health screening)	Weiner et al,^73^ 2022	Contextual factors integrated into care plan	OR = 2.67 (95% CI, 1.32-5.41)
Other (gun storage screening)	Sigel et al,^72^ 2023	Documenting gun storage counseling	51.2% vs 20.0%, *P* = .04
Other (substance use)	Lee et al,^54^ 2023	Alcohol intervention documented	57% vs 11%, *P* < .001
Routine health maintenance (wellness screening)	Lee et al,^54^ 2023	Alcohol screening documented	83.2% vs 20.8%, *P* < .001
Other (substance use)	Lee et al,^54^ 2023	Alcohol diagnoses	33.8% vs 28.8%, *P* = .003
Other (designating a proxy)	Loo et al,^38^ 2011	Designation of health care proxy	OR = 1.55 (95% CI, 1.00-2.41)
Other (social determinants of health screening)	Weiner et al,^63^ 2022	Improving or resolving “red flags”	OR = 0.96 (95% CI, 0.57-1.63)
Other (social determinants of health screening)	Weiner et al,^63^ 2022	Probing “red flags”	OR = 2.12 (95% CI, 1.14-3.93)

Abbreviations: ACE, angiotensin-converting enzyme inhibitors; ADHD, attention-deficit/hyperactivity disorder; ARB, angiotensin receptor blockers; CAD, coronary artery disease; CHF, coronary heart failure; CKD, chronic kidney disease; COPD, chronic obstructive pulmonary disease; DiD, difference-in-differences; eGFR, estimated glomerular filtration rate; EHR, electronic health records system; GERD, gastroesophageal reflux disease; HPV, human papillomavirus; HR, hazard ratio; IRR, incidence risk ratio; MRI, magnetic resonance imaging; NSAIDs, nonsteroidal anti-inflammatory drugs; OR, odds ratio.

^a^ All findings are reported with the EHR nudge first before the usual care. For studies reporting ratios, the comparator is usual care.

**Table 3.** Overall Findings of Association Between EHR Nudges and Patient Outcomes in Primary Care

Measure type and concept	Citation(s)	Measures	Overall findings^a^
**Patient-reported outcomes**			
Hypertension	Kressin et al,^67^ 2016	Antihypertensive adherence via self-reports	β = −0.34, *P* = .39
Other (pain management)	Dhingra et al,^74^ 2021	Pain intensity	Worst pain intensity scores: β = −0.50 (95% CI, −0.91 to −0.08); average pain intensity scores: β = −0.12 (95% CI, −0.57 to 0.33)
Other (pain management)	Dhingra et al,^74^ 2021	Pain interference	β = −1.33 (95% CI, −3.05 to 0.39)
**Patient adherence**			
Routine health maintenance (cancer screening)	Guiriguet et al,^75^ 2016	Colorectal cancer screening completed by patients	OR = 1.11 (95% CI, 1.02-1.22)
Hypertension	Tamblyn et al,^34^ 2018	Antihypertensive adherence via EHR data	Newly diagnosed uncomplicated hypertension: risk difference = 1.72 (95% CI, −6.91 to 10.36); established diagnoses: risk difference = 1.36 (95% CI, −1.55 to 4.28)
**Clinical outcomes**			
Other (hyperlipidemia)	Gill et al,^35^ 2009	At-goal lipid values	High-risk patients: OR = 1.17, *P* > .05; moderate-risk patients: OR = 0.29, *P* > .05; low-risk patients: OR = 1.74, *P* > .05
Hypertension	Abdel-Kader et al,2^5^ 2011	Blood pressure <130/80	OR = 2.07 (95% CI, 0.83-5.17)
Hypertension	Kr^es^sin et al,^67^ 2016	Diastolic blood pressure	β = −0.70, *P* = .06
Hypertension	Kressin et al,^67^ 2016	Systolic blood pressure	β = −0.62, *P* = .05
Other (atrial fibrillation)	Karlsson et al,^46^ 2018	Having stroke, transient ischemic attack, or systemic thromboembolism	49 vs 47, *P* = .64
Other (atrial fibrillation)	Karlsson et al,^46^ 2018	Having bleeding	12 vs 16, *P* = .04
Diabetes	Weiner et al,^63^ 2020	Hypoglycemia	5% vs 5%, *P* = .99

Abbreviations: EHR, electronic health records system; OR, odds ratio.

^a^ All findings are reported with the EHR nudge first before the usual care. For studies reporting ratios, the comparator is usual care.

### Study Quality Assessment Results

Overall, most studies (79.6%) were assessed to have a moderate risk of bias. For all cluster and parallel RCTs, there was limited measurement of clinicians’ adherence rate to the nudge intervention (ie, acknowledging the presence of the nudge by explicitly accepting or declining recommendations) in the final analysis. For crossover RCTs, there were concerns of short washout periods before administering the next phase. Across all RCTs, it was largely unclear whether outcome assessors were masked on study phases during the analysis and whether analytic methods used were predetermined prior to data collection. Some studies also experienced unexpected challenges in implementing or evaluating their EHR nudge as initially planned, such as hardware and software issues, concurrent quality improvement efforts, and changes in insurance coverage. Overall trends in study findings persisted even after removal of studies with high risk of bias (eTable 3 in Supplement 1).

### Health Care Quality

#### Patient Safety

Eleven studies together assessed 12 unique patient safety measures with all targeting appropriate medication prescribing patterns.^21,22,23,24,25,26,27,28,29,30,31^ Medications examined included antibiotics, opiates, anticholinergics, hypnotics, benzodiazepines, and nonsteroidal anti-inflammatory drugs (NSAIDs). Overall, studies reported mixed associations. EHR nudges were associated with improvements for 4 of the 12 measures. Another 2 measures were associated with improvements but under specific contexts (ie, disease type, nudge characteristics). For example, EHR nudges were associated with improvements in antibiotic prescribing for pharyngitis (−3.0% difference; *P* = .003) but not for urinary tract infections (−0.4% difference; *P* = .64). Nudges that included accountable justification mechanisms were associated with more appropriate prescribing compared with nudges that suggested alternatives.

#### Effectiveness

Thirty-seven studies together assessed 48 unique effectiveness measures.^24,25,32,33,34,35,36,37,38,39,40,41,42,43,44,45,46,47,48,49,50,51,52,53,54,55,56,57,58,59,60,61,62,63,64,65,66^ Clinical concepts included immunizations, wellness screening, cancer screening, and chronic care management. Overall, we observed mixed associations. EHR nudges were associated with improvements for 19 of the measures, such as increased rates of tetanus and pneumococcal immunizations; higher screening rates for hepatitis B and alcohol use; and providing guideline-concordant care for patients with heart failure or coronary artery disease. An additional 13 measures also were associated with improvements under specific contexts (eg, medical condition, patient risk level, time since diagnosis).

#### Patient-Centeredness

Two studies together assessed 3 unique patient-centeredness measures.^55,67^ Clinical concepts were limited to weight management and hypertension. Overall, EHR nudges were associated with improvements in counseling rates for exercise, diet, and blood pressure.

#### Timeliness

No study that we found assessed the association between EHR nudges and timeliness of care. Therefore, we were unable to assess for timeliness of care.

#### Efficiency

Four studies together assessed 4 unique efficiency measures in the context of reducing inappropriate or contraindicated laboratory tests and imaging.^26,48,68,69^ Overall, EHR nudges were not associated with improvements in any study.

#### Descriptive

Thirteen studies together assessed 38 unique descriptive measures.^25,37,38,44,45,49,50,54,55,70,71,72,73^ Overall, most studies found that EHR nudges were associated with improvements of various documentation and care planning patterns with fewer studies reporting null associations.

### Patient Outcomes

#### Patient-Reported Outcomes

Two studies together assessed 3 unique patient-reported outcome measures. These included measures for pain symptoms and self-reported medication adherence.^67,74^ EHR nudges were associated with improvements in pain intensity and null associations with medication adherence and pain interference.

#### Patient Adherence

Two studies together assessed 2 unique patient adherence measures using objective data sources (eg, EHR data).^34,75^ One study found that EHR nudges were associated with improved rates of completed colorectal screening kits by patients, whereas the other study found null association for antihypertensive adherence based on EHR data.

#### Clinical Outcomes

Five studies together assessed 7 unique clinical outcomes, including blood pressure, blood glucose, and hyperlipidemia levels as well as risk levels for bleeding among patients with atrial fibrillation and stroke.^25,35,46,63,67^ Overall, EHR nudges had null associations for most measures. However, EHR nudges were associated with improvements in the risk of bleeding among patients with atrial fibrillation.

### Implementation Facilitators and Barriers

Seven studies assessed clinicians’ viewpoints on implementation facilitators and barriers. Clinicians identified barriers, such as lack of knowledge or disagreement with guidelines and perceived errors in the programming of the nudge in the EHR (eTable 2 in Supplement 1).

## Discussion

This systematic review aimed to summarize available RCTs evaluating how EHR nudges were associated with health care quality and outcomes in primary care settings. Overall, our findings suggest that EHR nudges may improve specific dimensions of health care quality (ie, descriptive, patient-centeredness). There were less consistent findings about how EHR nudges were associated with other health care quality measures (eg, effectiveness) and patient outcomes. To our knowledge, no study has attempted to assess how EHR nudges were associated with timeliness of care.

We found that EHR nudges may be associated with modest improvements in descriptive measures of health care quality. This suggests that EHR nudges may facilitate improvements in clinicians’ documentation patterns, such as completeness or accuracy. Interestingly, this pattern was less consistent for studies targeting clinicians’ ordering behaviors. This difference may be, in part, due to differences in nudge mechanisms and uptake of the nudge. For instance, templates were more common among studies that aimed to improve documentation patterns compared with ordering behaviors. Although most studies in our review did not measure clinician adherence to the nudge, other studies have found that primary care clinicians have increasingly adopted templates to support their documentation (29% in 2007-2008 vs more than 90% in 2018-2020).^53,76^ This suggests that higher clinician acceptance of a nudge or its fit in the workflow may be important factors that influence the effectiveness of the nudge. Other factors may include implementation contexts and approaches. Few studies in our review attempted to assess implementation facilitators and barriers of their nudges. Thus, further qualitative research on clinicians’ attitudes and perceptions on current EHR nudges are needed to inform efforts on how to optimize the delivery of EHR nudges in primary care contexts.

In comparison to descriptive and patient-centeredness measures, other types of health care quality measures (ie, patient safety, effectiveness, efficiency) showed relatively less consistent improvement. Although there were several studies that suggested associations between EHR nudges and improved ordering behaviors, there were also just as many studies reporting null associations. One reason for these different findings may be that the effect varies across clinical conditions. For instance, most studies examining EHR nudges related to substance use revealed a pattern of improved outcomes. Another reason for these differences in findings may stem from the presence of additional factors, such as patient preferences and insurance coverage, that may influence whether orders are placed. One dimension of health care quality that has been relatively understudied is timeliness in primary care, revealing an area in need of additional research.

Our review found that most of the studies assessing patient outcomes assessed them within a 1-year timeframe, suggesting that additional studies measuring sustainability of EHR nudges are needed. Consequently, our findings should be interpreted as short-term outcomes. Like other systematic reviews of nudge interventions in and outside of the EHR,^10,11^ a majority of the studies in our review only examined short-term outcomes. Thus, the long-term outcomes of EHR nudges remain unclear. Furthermore, most of the available studies examined metabolic diseases (hyperlipidemia, hypertension, diabetes). Meanwhile, to our knowledge, no RCT has been conducted that targets other diseases that are treated and managed in primary care, such as depression, anxiety, chronic obstructive pulmonary disease, and asthma. Consequently, additional research is needed on more diverse types of patient outcomes and with longer evaluation periods.

Our systematic review revealed that there were only 11 studies (20.4%) that used the EHR nudge to facilitate a decrease in a targeted behavior, such as reducing antibiotic prescribing for viral infections, imaging orders for low back pain, and reducing overprescribing of hypnotics and psychotropic drugs. Together, these accounted for 11 unique measures. Only 1 of the 11 measures (9.1%) reported that EHR nudges were associated with a decrease in the targeted behavior (a decrease in inappropriate prescribing across a variety of conditions). In contrast, EHR nudges were associated with improvements for 52 of the 95 measures (54.7%) where the EHR nudge was designed and implemented to facilitate an increase in a targeted behavior. Due to the low number of available studies involving EHR nudges to deimplement specific practices, it may be premature to conclude that EHR nudges may be better suited for increasing behavior frequency. Nonetheless, it remains unclear what the reasons are for this potential difference between nudges designed to decrease a behavior compared with those designed to increase a behavior. There exists an ongoing need for additional studies examining EHR nudges with the intent to decrease a behavior as well as qualitative research on how EHR nudges could help overcome inertia of reducing, restricting, removing, or replacing low-value clinical practices. These findings may provide additional insights as to whether EHR nudges should be designed differently to deimplement a particular practice or whether different concurrent interventions (eg, audit and feedback, insurance payment structures) need to be in place.

### Limitations

This review has limitations. First, most studies targeted physicians or nurses. Furthermore, of the few studies that examined nonphysicians, combined samples of physicians and another clinician type were used. It remains unclear if different members of the care team respond differently to EHR nudges. Second, few studies were conducted in safety-net settings, such as federally qualified health centers, that may require different approaches to implementing EHR nudges due to lower available resources and less advanced EHR systems.^77^ Third, EHR nudges may be partly influenced by user interface design elements by EHR vendors. The most commonly used EHR was Epic, limiting generalizability to other EHR vendors. Fourth, most individual measures were assessed by only 1 study, precluding definitive conclusions on how EHR nudges are associated with performance in specific areas (eg, documenting allergies, blood pressure). Additionally, our quality appraisal revealed that none of the included studies controlled for adherence to evaluate EHR nudges. The observed outcomes of an intervention may be partly influenced by how often it is used, which makes the interpretation of findings challenging. For instance, it is unclear whether null results were due to low efficacy of the EHR nudge vs implementation issues of the nudge (eg, lack of awareness among clinicians of the nudge’s existence).

Based on these limitations, we discuss additional areas for future research. First, studies are needed that explicitly target other key members of the care team, such as social workers, pharmacists, and dietitians. Different care team members often have differing EHR workflows, which may influence how they respond to EHR nudges. Second, although outside of the scope of this review, patients can also be targeted by nudge interventions. Further studies are needed that assess how patients respond to nudges as well as studies that compare nudge effectiveness between patients and clinicians. Third, additional research is needed to assess potential facilitators and barriers that may be encountered when implementing EHR nudges in care settings. In a similar vein, there is a need for comparative evaluations of different EHR interfaces used to deliver the nudge to better understand what specific design elements facilitate the salience and effectiveness of the nudge. Additionally, because the number of studies that examined process measures outnumbered those that examined patient outcome measures, there persists a need for studies that assess how EHR nudges may influence downstream patient outcomes.

## Conclusions

EHR-based nudge interventions are a promising strategy for improving evidence-based care delivery in primary care, a setting that is critical for supporting population health. Findings from this systematic review suggest that nudges may change specific dimensions of health care quality, such as descriptive measures (ie, documentation patterns). Their associations with other dimensions and clinical outcomes have been less consistent. To strengthen the evidence for nudge interventions, additional implementation studies are needed to understand the context of when nudge interventions work.

## Notes

Supplement 1. **eTable 1.** Search Strategy **eTable 2.** Study Characteristics **eTable 3.** Study Quality Assessment Results

Supplement 2.
Data Sharing Statement
