# Error in Byline

**DOI:** 10.1001/jamanetworkopen.2025.58882

**Published:** 2026-01-22

In the Original Investigation titled “Electronic Health Record Nudges and Health Care Quality and Outcomes in Primary Care: A Systematic Review,”^1^ published September 17, 2024, Ms Katoju’s name was misspelled in the byline. This article has been corrected.^1^
