# Supplementary material for: Electronic Health Record Nudges and Health Care Quality and Outcomes in Primary Care: A Systematic Review
Source: JAMA Netw Open. 2024 Sep 17;7(9):e2432760. doi: 10.1001/jamanetworkopen.2024.32760 (PMC11409160; doi:10.1001/jamanetworkopen.2024.32760)
Supplement: Supplement 1. — eTable 1. Search Strategy eTable 2. Study Characteristics eTable 3. Study Quality Assessment Results [file jamanetwopen-e2432760-s001.pdf]

Supplemental Online Content

Nguyen OT, Kunta AR, Katoju SV, et al. Electronic health record nudges and health care quality and outcomes in primary care: a systematic review. *JAMA Netw Open.* 2024;7(9):e2432760. doi:10.1001/jamanetworkopen.2024.32760

eTable 1. Search Strategy

eTable 2. Study Characteristics

eTable 3. Study Quality Assessment Results

This supplemental material has been provided by the authors to give readers additional information about their work.

© 2024 Nguyen OT et al. *JAMA Network Open.*

eTable 1. Search strategy

Database	Terms	Results
MEDLINE/PubMed	MeSH Headings: (attending physicians, hospital OR osteopathic physicians OR internship and residency OR medical students OR primary care physicians OR medical staff OR physicians OR nursing OR nurses OR rehabilitation center OR allied health personnel OR allied health occupation OR physical therapy specialty OR occupational therapy OR speech therapy OR social work OR hospital chaplaincy service OR physician assistant) AND (((electronic health records[MeSH Terms:NoExp]) OR (medical record systems, computerized[MeSH Terms])) OR (clinical decision support systems[MeSH Terms]) OR (medical order entry system[MeSH Terms])	4,624

© 2024 Nguyen OT et al. *JAMA Network Open.*

Keywords: ("intern" OR "interns" OR "internship" OR "resident" OR "residents" OR "residency" OR "residencies" OR trainee* OR training OR fellow* OR postdoc* OR "post-doctoral" OR "post-doc*" OR student* OR hcp OR hcps OR "health care provider" OR "healthcare providers" OR clinician* OR "general practitioner" OR "general practitioners" OR "primary care provider" OR "primary care providers" OR "PCP" OR "PCPs" OR internist* OR "primary care physician" "primary care physicians" OR surge* OR "general practice" OR "general practitioner" OR "general practitioners" OR "health care professional" OR "health care professionals" OR psychiatri* OR hospitalist* OR "internal medicine" OR internist* OR cardiolog* OR radiolog* OR anesthe* OR anaesthe* OR pediatric* OR paediatric* OR geriatric* OR orthoped* OR orthopaed* OR ophthalmolog* OR neurolog* OR urolog* OR pulmo* OR oncolog* OR otolaryngolog* OR dermatolog* OR otorhinolaryngolog* OR gynecolog* OR gynaecolg* OR obsteric* OR gastroenterolog* OR endocrinolog* OR hepatolog* OR hematolog* OR neurosurge* OR patholog* OR rheumatolog* OR immunolog* OR "infectious disease" OR "infectious diseases" OR "communicable disease" OR "communicable diseases" OR nephrolog* OR "emergency medicine" OR "medical examiner" OR "medical examiners" OR coroner* OR osteopath* OR endodont* OR anatomist* OR "behavioral health" OR "behavioural health" OR "mental health" OR "health care practitioner" OR "health care practitioners" OR "healthcare practitioner" OR "healthcare practitioners" "care practitioner" OR "care practitioners" OR "health practitioner" OR "health practitioners" OR "home health" OR "MD" OR "MDs" OR "DO" OR "DOs" OR "attending physician" OR prescriber* OR "attending physicians" OR "physician" OR "physicians" OR doctor* OR "Rehabilitation therapist" OR "rehabilitation therapists" OR "rehabilitation therapy" OR "allied health" OR "physical therapy" OR "physical therapist" OR "physical therapists" OR "occupational therapy" OR "occupational therapist" OR "occupational therapists" OR "speech therapy" OR "speech therapist" OR "speech therapists" OR "social work" OR "social worker" OR "social workers" OR "chaplain" OR "chaplains" OR "physician assistant" OR "physician assistants" OR "physician associate" OR "physician associates" OR "midlevel provider" OR "mid-level provider" OR "midlevel providers" OR "midlevel provider" OR "physician extender" OR "physician extenders" OR "Nursing student" OR "nursing students" OR nurse* OR nursing OR midwi* OR doula* OR "registered nurse" OR "registered nurses" OR "licensed practical nurse" OR "licensed practical nurses" OR "licensed practical nursing" OR "nursing assistant" OR "birth assistant" OR "birth attendant" OR "nursing aide" OR "nursing aides" OR "geriatric aide" OR "advanced registered nurse practitioner" OR "advanced registered nurse practitioners" OR "advanced practice registered nurse" OR "advanced practice registered nurses" OR "RN" OR "RNs" OR "ARNP" OR "ARNPs" OR "LPN" OR "LPNs" OR "APRN" OR "APRNs" OR "nurse anesthetist" OR "nurse anesthetists" OR "advanced practice nurse" OR "advanced practice nurses" OR "advanced practice nursing" OR "cardiovascular nursing" OR "community health nursing" OR "critical care nursing" OR "emergency nursing" OR "critical care nursing" OR "emergency nursing" OR "ER nurse" OR "ER nurses" OR "ICU nurse" OR "ICU nurses" OR "ICU nursing" OR "family nursing" OR "forensic nursing" OR "geriatric nursing" OR "holistic nursing" OR "hospice nursing" OR "palliative care nursing" OR "maternal-child nursing" OR midwifery OR "military nursing" OR "nephrology nursing" OR "neuroscience nursing" OR "obstetric nursing" OR "occupational health nursing" OR "oncology nursing" OR "orthopedic nursing" OR "pediatric nursing" OR "perioperative nursing" OR "psychiatric nursing" OR "public health nursing" OR "radiologic nursing" OR "imaging nursing" OR "rehabilitation nursing" OR

"rural nursing" OR "school nursing" OR "transcultural nursing" OR "travelling nurse" OR "travel nurse") AND (“Choice architecture” OR default* OR feedback OR “opt-in” OR “opt-out” OR “active choice” OR “active choices” OR “automated alert” OR “automated alerts” OR “automated reminder” OR “automated reminders” OR fire* OR firing OR trigger* OR template* OR “structured documentation” OR SmartPhrase* OR auto-fill* OR autofill* OR preload* OR autopend* OR unanswered OR “standing order” OR “standing orders” OR predetermin* OR overrid* OR “behavioral economic” OR “behavioral economics” OR “behavioral economist” OR “behavioral economists” OR nudge*) AND (EHR OR EMR OR EHRs OR EMRs OR "electronic health record" OR "electronic health records" OR "electronic medical record" OR "electronic medical records" OR "computerized medical record" OR "computerized medical records" OR CDS OR “clinical decision support” OR CPOE OR “computerized physician order entry” OR “order entry” OR “computerized provider order entry” OR InBasket OR “In Basket” OR “patient messages” OR “patient-generated messages” OR “e-prescribing” OR “e-prescribe” OR “electronic prescription” OR “electronic prescriptions” OR “electronic prescribing”) AND (“choice architecture” OR default* OR feedback OR “opt-in” OR “opt-out” OR “active choice” OR “active choices” OR “automated alert” OR “automated alerts” OR “automated reminder” OR “automated reminders” OR fire* OR firing OR trigger* OR template* OR “structured documentation” OR SmartPhrase* OR auto-fill* OR autofill* OR preload* OR autopend* OR unanswered OR “standing order” OR “standing orders” OR predetermin* OR overrid* OR “behavioral economic” OR “behavioral economics” OR “behavioral economist” OR “behavioral economists” OR nudge*)

© 2024 Nguyen OT et al. *JAMA Network Open.*

Embase	Keywords: ("intern" OR "interns" OR "internship" OR "resident" OR "residents" OR "residency" OR "residencies" OR trainee* OR training OR fellow* OR postdoc* OR "post-doctoral" OR "post-doc*" OR student* OR hcp OR hcps OR "health care provider" OR "healthcare providers" OR clinician* OR "general practitioner" OR "general practitioners" OR "primary care provider" OR "primary care providers" OR "PCP" OR "PCPs" OR internist* OR "primary care physician" "primary care physicians" OR surge* OR "general practice" OR "general practitioner" OR "general practitioners" OR "health care professional" OR "health care professionals" OR psychiatri* OR hospitalist* OR "internal medicine" OR internist* OR cardiolog* OR radiolog* OR anesthe* OR anaesthe* OR pediatric* OR paediatric* OR geriatric* OR orthoped* OR orthopaed* OR ophthalmolog* OR neurolog* OR urolog* OR pulmo* OR oncolog* OR otolaryngolog* OR dermatolog* OR otorhinolaryngolog* OR gynecolog* OR gynaecolg* OR obsteric* OR gastroenterolog* OR endocrinolog* OR hepatolog* OR hematolog* OR neurosurge* OR patholog* OR rheumatolog* OR immunolog* OR "infectious disease" OR "infectious diseases" OR "communicable disease" OR "communicable diseases" OR nephrolog* OR "emergency medicine" OR "medical examiner" OR "medical examiners" OR coroner* OR osteopath* OR endodont* OR anatomist* OR "behavioral health" OR "behavioural health" OR "mental health" OR "health care practitioner" OR "health care practitioners" OR "healthcare practitioner" OR "healthcare practitioners" "care practitioner" OR "care practitioners" OR "health practitioner" OR "health practitioners" OR "home health" OR "MD" OR "MDs" OR "DO" OR "DOs" OR "attending physician" OR prescriber* OR "attending physicians" OR "physician" OR "physicians" OR doctor* OR "Rehabilitation therapist" OR "rehabilitation therapists" OR "rehabilitation therapy" OR "allied health" OR "physical therapy" OR "physical therapist" OR "physical therapists" OR "occupational therapy" OR "occupational therapist" OR "occupational therapists" OR "speech therapy" OR "speech therapist" OR "speech therapists" OR "social work" OR "social worker" OR "social workers" OR "chaplain" OR "chaplains" OR "physician assistant" OR "physician assistants" OR "physician associate" OR "physician associates" OR "midlevel provider" OR "mid-level provider" OR "midlevel providers" OR "midlevel provider" OR "physician extender" OR "physician extenders" OR "Nursing student" OR "nursing students" OR nurse* OR nursing OR midwi* OR doula* OR "registered nurse" OR "registered nurses" OR "licensed practical nurse" OR "licensed practical nurses" OR "licensed practical nursing" OR "nursing assistant" OR "birth assistant" OR "birth attendant" OR "nursing aide" OR "nursing aides" OR "geriatric aide" OR "advanced registered nurse practitioner" OR "advanced registered nurse practitioners" OR "advanced practice registered nurse" OR "advanced practice registered nurses" OR "RN" OR "RNs" OR "ARNP" OR "ARNPs" OR "LPN" OR "LPNs" OR "APRN" OR "APRNs" OR "nurse anesthetist" OR "nurse anesthetists" OR "advanced practice nurse" OR "advanced practice nurses" OR "advanced practice nursing" OR "cardiovascular nursing" OR "community health nursing" OR "critical care nursing" OR "emergency nursing" OR "critical care nursing" OR "emergency nursing" OR "ER nurse" OR "ER nurses" OR "ICU nurse" OR "ICU nurses" OR "ICU nursing" OR "family nursing" OR "forensic nursing" OR "geriatric nursing" OR "holistic nursing" OR "hospice nursing" OR "palliative care nursing" OR "maternal-child nursing" OR midwifery OR "military nursing" OR "nephrology nursing" OR "neuroscience nursing" OR "obstetric nursing" OR "occupational health nursing" OR "oncology nursing" OR "orthopedic nursing" OR "pediatric nursing" OR "perioperative nursing" OR "psychiatric nursing" OR "public health nursing" OR "radiologic nursing" OR "imaging nursing" OR "rehabilitation nursing" OR	10,926

© 2024 Nguyen OT et al. *JAMA Network Open.*

"rural nursing" OR "school nursing" OR "transcultural nursing" OR "travelling nurse" OR "travel nurse") AND (“Choice architecture” OR default* OR feedback OR “opt-in” OR “opt-out” OR “active choice” OR “active choices” OR “automated alert” OR “automated alerts” OR “automated reminder” OR “automated reminders” OR fire* OR firing OR trigger* OR template* OR “structured documentation” OR SmartPhrase* OR auto-fill* OR autofill* OR preload* OR autopend* OR unanswered OR “standing order” OR “standing orders” OR predetermin* OR overrid* OR “behavioral economic” OR “behavioral economics” OR “behavioral economist” OR “behavioral economists” OR nudge*) AND (EHR OR EMR OR EHRs OR EMRs OR "electronic health record" OR "electronic health records" OR "electronic medical record" OR "electronic medical records" OR "computerized medical record" OR "computerized medical records" OR CDS OR “clinical decision support” OR CPOE OR “computerized physician order entry” OR “order entry” OR “computerized provider order entry” OR InBasket OR “In Basket” OR “patient messages” OR “patient-generated messages” OR “e-prescribing” OR “e-prescribe” OR “electronic prescription” OR “electronic prescriptions” OR “electronic prescribing”) AND (“choice architecture” OR default* OR feedback OR “opt-in” OR “opt-out” OR “active choice” OR “active choices” OR “automated alert” OR “automated alerts” OR “automated reminder” OR “automated reminders” OR fire* OR firing OR trigger* OR template* OR “structured documentation” OR SmartPhrase* OR auto-fill* OR autofill* OR preload* OR autopend* OR unanswered OR “standing order” OR “standing orders” OR predetermin* OR overrid* OR “behavioral economic” OR “behavioral economics” OR “behavioral economist” OR “behavioral economists” OR nudge*)

© 2024 Nguyen OT et al. *JAMA Network Open.*

PsycINFO	Keywords: ("intern" OR "interns" OR "internship" OR "resident" OR "residents" OR "residency" OR "residencies" OR trainee* OR training OR fellow* OR postdoc* OR "post-doctoral" OR "post-doc*" OR student* OR hcp OR hcps OR "health care provider" OR "healthcare providers" OR clinician* OR "general practitioner" OR "general practitioners" OR "primary care provider" OR "primary care providers" OR "PCP" OR "PCPs" OR internist* OR "primary care physician" "primary care physicians" OR surge* OR "general practice" OR "general practitioner" OR "general practitioners" OR "health care professional" OR "health care professionals" OR psychiatri* OR hospitalist* OR "internal medicine" OR internist* OR cardiolog* OR radiolog* OR anesthe* OR anaesthe* OR pediatric* OR paediatric* OR geriatric* OR orthoped* OR orthopaed* OR ophthalmolog* OR neurolog* OR urolog* OR pulmo* OR oncolog* OR otolaryngolog* OR dermatolog* OR otorhinolaryngolog* OR gynecolog* OR gynaecolg* OR obsteric* OR gastroenterolog* OR endocrinolog* OR hepatolog* OR hematolog* OR neurosurge* OR patholog* OR rheumatolog* OR immunolog* OR "infectious disease" OR "infectious diseases" OR "communicable disease" OR "communicable diseases" OR nephrolog* OR "emergency medicine" OR "medical examiner" OR "medical examiners" OR coroner* OR osteopath* OR endodont* OR anatomist* OR "behavioral health" OR "behavioural health" OR "mental health" OR "health care practitioner" OR "health care practitioners" OR "healthcare practitioner" OR "healthcare practitioners" "care practitioner" OR "care practitioners" OR "health practitioner" OR "health practitioners" OR "home health" OR "MD" OR "MDs" OR "DO" OR "DOs" OR "attending physician" OR prescriber* OR "attending physicians" OR "physician" OR "physicians" OR doctor* OR "Rehabilitation therapist" OR "rehabilitation therapists" OR "rehabilitation therapy" OR "allied health" OR "physical therapy" OR "physical therapist" OR "physical therapists" OR "occupational therapy" OR "occupational therapist" OR "occupational therapists" OR "speech therapy" OR "speech therapist" OR "speech therapists" OR "social work" OR "social worker" OR "social workers" OR "chaplain" OR "chaplains" OR "physician assistant" OR "physician assistants" OR "physician associate" OR "physician associates" OR "midlevel provider" OR "mid-level provider" OR "midlevel providers" OR "midlevel provider" OR "physician extender" OR "physician extenders" OR "Nursing student" OR "nursing students" OR nurse* OR nursing OR midwi* OR doula* OR "registered nurse" OR "registered nurses" OR "licensed practical nurse" OR "licensed practical nurses" OR "licensed practical nursing" OR "nursing assistant" OR "birth assistant" OR "birth attendant" OR "nursing aide" OR "nursing aides" OR "geriatric aide" OR "advanced registered nurse practitioner" OR "advanced registered nurse practitioners" OR "advanced practice registered nurse" OR "advanced practice registered nurses" OR "RN" OR "RNs" OR "ARNP" OR "ARNPs" OR "LPN" OR "LPNs" OR "APRN" OR "APRNs" OR "nurse anesthetist" OR "nurse anesthetists" OR "advanced practice nurse" OR "advanced practice nurses" OR "advanced practice nursing" OR "cardiovascular nursing" OR "community health nursing" OR "critical care nursing" OR "emergency nursing" OR "critical care nursing" OR "emergency nursing" OR "ER nurse" OR "ER nurses" OR "ICU nurse" OR "ICU nurses" OR "ICU nursing" OR "family nursing" OR "forensic nursing" OR "geriatric nursing" OR "holistic nursing" OR "hospice nursing" OR "palliative care nursing" OR "maternal-child nursing" OR midwifery OR "military nursing" OR "nephrology nursing" OR "neuroscience nursing" OR "obstetric nursing" OR "occupational health nursing" OR "oncology nursing" OR "orthopedic nursing" OR "pediatric nursing" OR "perioperative nursing" OR "psychiatric nursing" OR "public health nursing" OR "radiologic nursing" OR "imaging nursing" OR "rehabilitation nursing" OR	661

© 2024 Nguyen OT et al. *JAMA Network Open.*

"rural nursing" OR "school nursing" OR "transcultural nursing" OR "travelling nurse" OR "travel nurse") AND "Choice architecture" OR default* OR feedback OR "opt-in" OR "opt-out" OR "active choice" OR "active choices" OR "automated alert" OR "automated alerts" OR "automated reminder" OR "automated reminders" OR fire* OR firing OR trigger* OR template* OR "structured documentation" OR SmartPhrase* OR auto-fill* OR autofill* OR preload* OR autopend* OR unanswered OR "standing order" OR "standing orders" OR predetermin* OR overrid* OR "behavioral economic" OR "behavioral economics" OR "behavioral economist" OR "behavioral economists" OR nudge*) AND (EHR OR EMR OR EHRs OR EMRs OR "electronic health record" OR "electronic health records" OR "electronic medical record" OR "electronic medical records" OR "computerized medical record" OR "computerized medical records" OR CDS OR "clinical decision support" OR CPOE OR "computerized physician order entry" OR "order entry" OR "computerized provider order entry" OR InBasket OR "In Basket" OR "patient messages" OR "patient-generated messages" OR "e-prescribing" OR "e-prescribe" OR "electronic prescription" OR "electronic prescriptions" OR "electronic prescribing") AND ("choice architecture" OR default* OR feedback OR "opt-in" OR "opt-out" OR "active choice" OR "active choices" OR "automated alert" OR "automated alerts" OR "automated reminder" OR "automated reminders" OR fire* OR firing OR trigger* OR template* OR "structured documentation" OR SmartPhrase* OR auto-fill* OR autofill* OR preload* OR autopend* OR unanswered OR "standing order" OR "standing orders" OR predetermin* OR overrid* OR "behavioral economic" OR "behavioral economics" OR "behavioral economist" OR "behavioral economists" OR nudge*)

© 2024 Nguyen OT et al. *JAMA Network Open.*

CINAHL	Keywords: ("intern" OR "interns" OR "internship" OR "resident" OR "residents" OR "residency" OR "residencies" OR trainee* OR training OR fellow* OR postdoc* OR "post-doctoral" OR "post-doc*" OR student* OR hcp OR hcps OR "health care provider" OR "healthcare providers" OR clinician* OR "general practitioner" OR "general practitioners" OR "primary care provider" OR "primary care providers" OR "PCP" OR "PCPs" OR internist* OR "primary care physician" "primary care physicians" OR surge* OR "general practice" OR "general practitioner" OR "general practitioners" OR "health care professional" OR "health care professionals" OR psychiatri* OR hospitalist* OR "internal medicine" OR internist* OR cardiolog* OR radiolog* OR anesthe* OR anaesthe* OR pediatric* OR paediatric* OR geriatric* OR orthoped* OR orthopaed* OR ophthalmolog* OR neurolog* OR urolog* OR pulmo* OR oncolog* OR otolaryngolog* OR dermatolog* OR otorhinolaryngolog* OR gynecolog* OR gynaecolg* OR obsteric* OR gastroenterolog* OR endocrinolog* OR hepatolog* OR hematolog* OR neurosurge* OR patholog* OR rheumatolog* OR immunolog* OR "infectious disease" OR "infectious diseases" OR "communicable disease" OR "communicable diseases" OR nephrolog* OR "emergency medicine" OR "medical examiner" OR "medical examiners" OR coroner* OR osteopath* OR endodont* OR anatomist* OR "behavioral health" OR "behavioural health" OR "mental health" OR "health care practitioner" OR "health care practitioners" OR "healthcare practitioner" OR "healthcare practitioners" "care practitioner" OR "care practitioners" OR "health practitioner" OR "health practitioners" OR "home health" OR "MD" OR "MDs" OR "DO" OR "DOs" OR "attending physician" OR prescriber* OR "attending physicians" OR "physician" OR "physicians" OR doctor* OR "Rehabilitation therapist" OR "rehabilitation therapists" OR "rehabilitation therapy" OR "allied health" OR "physical therapy" OR "physical therapist" OR "physical therapists" OR "occupational therapy" OR "occupational therapist" OR "occupational therapists" OR "speech therapy" OR "speech therapist" OR "speech therapists" OR "social work" OR "social worker" OR "social workers" OR "chaplain" OR "chaplains" OR "physician assistant" OR "physician assistants" OR "physician associate" OR "physician associates" OR "midlevel provider" OR "mid-level provider" OR "midlevel providers" OR "midlevel provider" OR "physician extender" OR "physician extenders" OR "Nursing student" OR "nursing students" OR nurse* OR nursing OR midwi* OR doula* OR "registered nurse" OR "registered nurses" OR "licensed practical nurse" OR "licensed practical nurses" OR "licensed practical nursing" OR "nursing assistant" OR "birth assistant" OR "birth attendant" OR "nursing aide" OR "nursing aides" OR "geriatric aide" OR "advanced registered nurse practitioner" OR "advanced registered nurse practitioners" OR "advanced practice registered nurse" OR "advanced practice registered nurses" OR "RN" OR "RNs" OR "ARNP" OR "ARNPs" OR "LPN" OR "LPNs" OR "APRN" OR "APRNs" OR "nurse anesthetist" OR "nurse anesthetists" OR "advanced practice nurse" OR "advanced practice nurses" OR "advanced practice nursing" OR "cardiovascular nursing" OR "community health nursing" OR "critical care nursing" OR "emergency nursing" OR "critical care nursing" OR "emergency nursing" OR "ER nurse" OR "ER nurses" OR "ICU nurse" OR "ICU nurses" OR "ICU nursing" OR "family nursing" OR "forensic nursing" OR "geriatric nursing" OR "holistic nursing" OR "hospice nursing" OR "palliative care nursing" OR "maternal-child nursing" OR midwifery OR "military nursing" OR "nephrology nursing" OR "neuroscience nursing" OR "obstetric nursing" OR "occupational health nursing" OR "oncology nursing" OR "orthopedic nursing" OR "pediatric nursing" OR "perioperative nursing" OR "psychiatric nursing" OR "public health nursing" OR "radiologic nursing" OR "imaging nursing" OR "rehabilitation nursing" OR	2,703

"rural nursing" OR "school nursing" OR "transcultural nursing" OR "travelling nurse" OR "travel nurse") AND (“Choice architecture” OR default* OR feedback OR “opt-in” OR “opt-out” OR “active choice” OR “active choices” OR “automated alert” OR “automated alerts” OR “automated reminder” OR “automated reminders” OR fire* OR firing OR trigger* OR template* OR “structured documentation” OR SmartPhrase* OR auto-fill* OR autofill* OR preload* OR autopend* OR unanswered OR “standing order” OR “standing orders” OR predetermin* OR overrid* OR “behavioral economic” OR “behavioral economics” OR “behavioral economist” OR “behavioral economists” OR nudge*) AND (EHR OR EMR OR EHRs OR EMRs OR "electronic health record" OR "electronic health records" OR "electronic medical record" OR "electronic medical records" OR "computerized medical record" OR "computerized medical records" OR CDS OR “clinical decision support” OR CPOE OR “computerized physician order entry” OR “order entry” OR “computerized provider order entry” OR InBasket OR “In Basket” OR “patient messages” OR “patient-generated messages” OR “e-prescribing” OR “e-prescribe” OR “electronic prescription” OR “electronic prescriptions” OR “electronic prescribing”) AND (“choice architecture” OR default* OR feedback OR “opt-in” OR “opt-out” OR “active choice” OR “active choices” OR “automated alert” OR “automated alerts” OR “automated reminder” OR “automated reminders” OR fire* OR firing OR trigger* OR template* OR “structured documentation” OR SmartPhrase* OR auto-fill* OR autofill* OR preload* OR autopend* OR unanswered OR “standing order” OR “standing orders” OR predetermin* OR overrid* OR “behavioral economic” OR “behavioral economics” OR “behavioral economist” OR “behavioral economists” OR nudge*)

© 2024 Nguyen OT et al. *JAMA Network Open.*

Web of Science	Keywords: ("intern" OR "interns" OR "internship" OR "resident" OR "residents" OR "residency" OR "residencies" OR trainee* OR training OR fellow* OR postdoc* OR "post-doctoral" OR "post-doc*" OR student* OR hcp OR hcps OR "health care provider" OR "healthcare providers" OR clinician* OR "general practitioner" OR "general practitioners" OR "primary care provider" OR "primary care providers" OR "PCP" OR "PCPs" OR internist* OR "primary care physician" "primary care physicians" OR surge* OR "general practice" OR "general practitioner" OR "general practitioners" OR "health care professional" OR "health care professionals" OR psychiatri* OR hospitalist* OR "internal medicine" OR internist* OR cardiolog* OR radiolog* OR anesthe* OR anaesthe* OR pediatric* OR paediatric* OR geriatric* OR orthoped* OR orthopaed* OR ophthalmolog* OR neurolog* OR urolog* OR pulmo* OR oncolog* OR otolaryngolog* OR dermatolog* OR otorhinolaryngolog* OR gynecolog* OR gynaecolg* OR obsteric* OR gastroenterolog* OR endocrinolog* OR hepatolog* OR hematolog* OR neurosurge* OR patholog* OR rheumatolog* OR immunolog* OR "infectious disease" OR "infectious diseases" OR "communicable disease" OR "communicable diseases" OR nephrolog* OR "emergency medicine" OR "medical examiner" OR "medical examiners" OR coroner* OR osteopath* OR endodont* OR anatomist* OR "behavioral health" OR "behavioural health" OR "mental health" OR "health care practitioner" OR "health care practitioners" OR "healthcare practitioner" OR "healthcare practitioners" "care practitioner" OR "care practitioners" OR "health practitioner" OR "health practitioners" OR "home health" OR "MD" OR "MDs" OR "DO" OR "DOs" OR "attending physician" OR prescriber* OR "attending physicians" OR "physician" OR "physicians" OR doctor* OR "Rehabilitation therapist" OR "rehabilitation therapists" OR "rehabilitation therapy" OR "allied health" OR "physical therapy" OR "physical therapist" OR "physical therapists" OR "occupational therapy" OR "occupational therapist" OR "occupational therapists" OR "speech therapy" OR "speech therapist" OR "speech therapists" OR "social work" OR "social worker" OR "social workers" OR "chaplain" OR "chaplains" OR "physician assistant" OR "physician assistants" OR "physician associate" OR "physician associates" OR "midlevel provider" OR "mid-level provider" OR "midlevel providers" OR "midlevel provider" OR "physician extender" OR "physician extenders" OR "Nursing student" OR "nursing students" OR nurse* OR nursing OR midwi* OR doula* OR "registered nurse" OR "registered nurses" OR "licensed practical nurse" OR "licensed practical nurses" OR "licensed practical nursing" OR "nursing assistant" OR "birth assistant" OR "birth attendant" OR "nursing aide" OR "nursing aides" OR "geriatric aide" OR "advanced registered nurse practitioner" OR "advanced registered nurse practitioners" OR "advanced practice registered nurse" OR "advanced practice registered nurses" OR "RN" OR "RNs" OR "ARNP" OR "ARNPs" OR "LPN" OR "LPNs" OR "APRN" OR "APRNs" OR "nurse anesthetist" OR "nurse anesthetists" OR "advanced practice nurse" OR "advanced practice nurses" OR "advanced practice nursing" OR "cardiovascular nursing" OR "community health nursing" OR "critical care nursing" OR "emergency nursing" OR "critical care nursing" OR "emergency nursing" OR "ER nurse" OR "ER nurses" OR "ICU nurse" OR "ICU nurses" OR "ICU nursing" OR "family nursing" OR "forensic nursing" OR "geriatric nursing" OR "holistic nursing" OR "hospice nursing" OR "palliative care nursing" OR "maternal-child nursing" OR midwifery OR "military nursing" OR "nephrology nursing" OR "neuroscience nursing" OR "obstetric nursing" OR "occupational health nursing" OR "oncology nursing" OR "orthopedic nursing" OR "pediatric nursing" OR "perioperative nursing" OR "psychiatric nursing" OR "public health nursing" OR "radiologic nursing" OR "imaging nursing" OR "rehabilitation nursing" OR	5,202

© 2024 Nguyen OT et al. *JAMA Network Open.*

"rural nursing" OR "school nursing" OR "transcultural nursing" OR "travelling nurse" OR "travel nurse") AND (“Choice architecture” OR default* OR feedback OR “opt-in” OR “opt-out” OR “active choice” OR “active choices” OR “automated alert” OR “automated alerts” OR “automated reminder” OR “automated reminders” OR fire* OR firing OR trigger* OR template* OR “structured documentation” OR SmartPhrase* OR auto-fill* OR autofill* OR preload* OR autopend* OR unanswered OR “standing order” OR “standing orders” OR predetermin* OR overrid* OR “behavioral economic” OR “behavioral economics” OR “behavioral economist” OR “behavioral economists” OR nudge*) AND (EHR OR EMR OR EHRs OR EMRs OR "electronic health record" OR "electronic health records" OR "electronic medical record" OR "electronic medical records" OR "computerized medical record" OR "computerized medical records" OR CDS OR “clinical decision support” OR CPOE OR “computerized physician order entry” OR “order entry” OR “computerized provider order entry” OR InBasket OR “In Basket” OR “patient messages” OR “patient-generated messages” OR “e-prescribing” OR “e-prescribe” OR “electronic prescription” OR “electronic prescriptions” OR “electronic prescribing”) AND (“choice architecture” OR default* OR feedback OR “opt-in” OR “opt-out” OR “active choice” OR “active choices” OR “automated alert” OR “automated alerts” OR “automated reminder” OR “automated reminders” OR fire* OR firing OR trigger* OR template* OR “structured documentation” OR SmartPhrase* OR auto-fill* OR autofill* OR preload* OR autopend* OR unanswered OR “standing order” OR “standing orders” OR predetermin* OR overrid* OR “behavioral economic” OR “behavioral economics” OR “behavioral economist” OR “behavioral economists” OR nudge*)

© 2024 Nguyen OT et al. *JAMA Network Open.*

eTable 2. Study characteristics

Study	Country	Clinician Types Involved	Medical Conditions Studied	Length of Evaluation Period	Study Design	Sample Size	Overall Nudge Intent	Education Provided?	Supporting Materials (e.g., notecards, emailed screenshots) Provided to Clinicians?	Posters Placed in Clinic?	Auditing and Feedback Done?	Patient Education Material or Outreach Provided?	Had Guiding Choice Through Defaults?	Had Enabling Choice?	Had Framing Information?	Had Providing Information?	Electronic Health Records Vendor	Had Alert?	Had Template?	Had Order Set?	Implementation Facilitators	Implementation Barriers
McDowell et al., 1989	Canada	Physicians, nurses	Hypertension screening	1 year	Parallel RCT	Letter reminder: 1,508 patients Telephone reminder: 1,433 patients Computerized reminders: 1,432 patients Usual care: 1,371 patients	Increase behavior frequency	No	No	No	No	Yes	No	No	No	Yes	Not reported	Yes	No	No	Not assessed	Not assessed

© 2024 Nguyen OT et al. *JAMA Network Open.*

Flottorp et al., 2002	Norway	Not reported	Urinary tract infections, pharyngitis	4 months	Cluster RCT	Sore throat 56 intervention clinics, 57 control clinics<br><br>UTI 57 intervention clinics, 56 control clinics	Increase behavior frequency	Yes	Yes	No	No	Yes	No	No	No	Yes	WinMed	Yes	No	No	Not assessed	Not assessed
Tamblyn et al., 2003	Canada	Physicians	Overprescribing of medications	13 months	Cluster RCT	54 intervention physicians, 53 control physicians	Decrease behavior frequency	No	No	No	No	No	No	No	No	Yes	Not reported	Yes	No	No	None reported	Computer problems
Frank et al., 2004	Australia	Physicians	Multiple preventive screening and immunizations	Not applicable	Parallel RCT	Intervention group 5118 patients; control group 5389 patients	Increase behavior frequency	No	No	No	No	No	No	No	No	Yes	Not reported	Yes	No	No	Not assessed	Not assessed

© 2024 Nguyen OT et al. *JAMA Network Open.*

Sequist et al., 2005	United States	Physicians	Diabetes, coronary artery disease	6 months	Cluster RCT	92 physicians and 2,924 patients in intervention arm, 102 physicians and 3,319 patients in control arm	Increase behavior frequency	No	No	No	No	No	No	No	No	Yes	LMR	Yes	No	No	None reported	Lack of time during visit, patient noncompliance or refusal of treatment; lack of knowledge of specific guideline recommendation, lack of awareness of guideline's existence, disgree with guideline recommendation
Palen et al., 2006	United States	Physicians	Laboratory values monitoring	1 year	Parallel RCT	207 physicians total: Alert condition: 103; Usual care condition: 104	Increase behavior frequency	Yes	No	No	No	No	No	No	No	Yes	IBM - Clinical Information System	Yes	No	No	Not assessed	Not assessed

© 2024 Nguyen OT et al. *JAMA Network Open.*

						physiicans total																
Feldstein et al., 2006	United States	Not reported	Osteoporosis	25 day follow-up	Parallel RCT	15 total clinics: 1) EHR alert group (4 clinics, 44 clinicians, 196 patients); 2) voice mail group (3 clinics, 44 clinicians, 267 patients); 3) pharmacy team outreach (4 clinics, 56 clinicians, 261 patients); 4) Usual care group (4 clinics, 56 clinician	Increase behavior frequency	No	No	No	No	No	No	No	No	Yes	Not reported	Yes	Yes	No	Not assessed	Not assessed

© 2024 Nguyen OT et al. *JAMA Network Open.*

						s, 237 patients);																
van Wyk et al., 2008	Netherlands	Physicians	Dyslipidemia	12 month follow-up	Cluster RCT	Total: 38 practices; 1) Alert condition (13 practices; 26 physicians; 31211 patients); 2) On-demand support condition (14 practices; 34 physicians; 29518 patients); 3) Control condition (11 practices; 20 physicians; 31325 patients)	Increase behavior frequency	No	No	No	No	No	No	No	No	Yes	ELIAS system	Yes	No	No	Not assessed	Not assessed

© 2024 Nguyen OT et al. *JAMA Network Open.*

Lo et al., 2009	United States	Physicians, NPs, PAs, nurses	Laboratory values monitoring	Not applicable	Cluster RCT	Control: 191 providers and 1520 patients; intervention: 175 providers and 1245 patients	Increase behavior frequency	No	No	No	No	No	No	No	No	Yes	Longitudinal Medical Record	Yes	No	No	Not assessed	Not assessed
Gill et al., 2009	United States	Physicians	Lipids monitoring	1 year	Cluster RCT	12 offices intervention arm (26,996 patients, 49 physicians), 13 offices control arm (37,454 patients, 56 physicians)	Increase behavior frequency	No	No	No	No	Yes	No	No	No	Yes	Centricity	Yes	Yes	Yes	Not assessed	Not assessed
Linder et al., 2009	United States	Physicians, PAs, NPs	Smoking cessation	9 months	Cluster RCT	14 practices, 314 clinicians, and 68,309 patients in	Increase behavior frequency	No	No	No	No	No	No	No	No	Yes	LMR	Yes	No	Yes	Not assessed	Not assessed

© 2024 Nguyen OT et al. *JAMA Network Open.*

						control arm and 12 practices, 64,321 patients, and 207 clinicians in intervention arm																
Fortuna et al., 2009	United States	Physicians	Overprescribing of hypnotic medications	1 year	Cluster RCT	14 internal medicine practices<br><br>5 sites (80 clinicians) assigned to receive only alerts<br><br>4 sites (115 clinicians) assigned to receive both alerts	Decrease behavior frequency	Yes	Yes	No	No	No	No	Yes	No	Yes	Epic	Yes	Yes	Yes	Not assessed	Not assessed

© 2024 Nguyen OT et al. *JAMA Network Open.*

						and educational sessions)<br><br>5 sites (62 clinicians) assigned to usual care															
Schriefer et al., 2009	United States	Physicians	Obesity	Unclear	Cluster RCT	2 teams, 18 physicians, 379 patients were in intervention arm, 2 teams, 19 physicians, and 467 patients were in control arm	Increase behavior frequency	No	No	No	No	No	No	No	No	Yes	Not reported	Yes	No	No	Not assessed	Not assessed
Fiks et al., 2009	United States	Not reported	Influenza immunizations	6 months	Cluster RCT	5,809 patients in control arm, 6,110	Increase behavior frequency	Yes	Yes	No	No	No	No	No	No	Yes	Epic	Yes	No	No	Not assessed	Not assessed

© 2024 Nguyen OT et al. *JAMA Network Open.*

						patients in intervention arm																
Co et al., 2010	United States	Physicians	Attention-deficit/hyperactivity disorder	Not applicable	Cluster RCT	412 patients total: Intervention: 206 patients; control: 206 patients; each group included 3 community health centers and 3 private practices	Increase behavior frequency	Yes	Yes	No	No	No	No	No	Yes	Yes	Longitudinal Medical Record	Yes	Yes	No	Not assessed	Not assessed
Player et al., 2010	United States	Physicians, PAs, NPs	Gastroesophageal reflux disease	1 year	Cluster RCT	13 offices with 53 providers were randomized to the intervention group, and 14	Increase behavior frequency	Yes	Yes	No	No	Yes	No	Yes	No	Yes	Centricity	No	Yes	Yes	Not assessed	Not assessed

© 2024 Nguyen OT et al. *JAMA Network Open.*

						offices with 66 providers were randomized to the control group.																
Bell et al.,2010	United States	Physicians	Asthma	1 year	Cluster RCT	Control-9035 Intervention-10415 Total=19450	Increase behavior frequency	Yes	No	No	No	No	No	No	No	Yes	Epic	Yes	Yes	Yes	Not assessed	Not assessed
Gill et al., 2011	United States	Physicians, NPs, PAs	Gastrointestinal monitoring for patients taking non-steroidal anti-inflammatory drugs	1 year	Cluster RCT	53 clinicians and 2,222 patients in intervention arm, 66 clinicians and 3,012 patients in control arm	Increase behavior frequency, decrease behavior frequency	Yes	Yes	No	No	No	No	No	No	Yes	Centricity	Yes	Yes	Yes	Not reported	Took too much time
O'Connor et	United States	Physicians	Diabetes	21 months	Cluster RCT	6 clinics, 20 physicians,	Increase behavior	Yes	No	No	No	No	No	Yes	No	Yes	Not reported	No	No	No	Not assessed	Not assessed

© 2024 Nguyen OT et al. *JAMA Network Open.*

al., 2011						1,194 patients in intervention arm; 5 clinics, 20 physicians, 1,362 patients in control arm	frequency															
Abdel-Kader et al., 2011	United States	Physicians	Chronic kidney disease	1 year	Parallel RCT	103 patients and 15 physicians in control arm, 145 patients and 15 physicians in intervention arm	Increase behavior frequency, decrease behavior frequency	Yes	No	No	No	No	No	No	No	Yes	Epic	Yes	No	Yes	Not assessed	Not assessed
Loo et al., 2011	United States	Physicians	Pneumococcal immunizations, influenza immunizations, osteoporosis screening, designation	12 months	Parallel RCT	17 physicians and 1,336 patients received only EMR reminders, 17	Increase behavior frequency	No	No	No	No	No	No	No	No	Yes	Not reported	Yes	No	No	Not assessed	Not assessed

© 2024 Nguyen OT et al. *JAMA Network Open.*

			of a health care proxy			physicians and 1,394 patients received EMR reminders and panel management, 20 physicians and 1,930 patients in usual care																
Feder et al., 2011	United Kingdom	Not reported	Domestic violence screening	1 year	Cluster RCT	24 intervention clinics, 24 control clinics	Increase behavior frequency	Yes	Yes	Yes	Yes	No	No	No	No	Yes	Not reported	No	Yes	No	Not assessed	Not assessed
Tang et al., 2012	United States	Physicians	Weight management	6 months	Cluster RCT	15 intervention physicians, 15 control physicians. 200 randomly selected patients	Increase behavior frequency	Yes	No	No	No	No	No	No	Yes	Yes	Epic	Yes	Yes	Yes	Not reported	Insufficient time in clinic, tool complexity, patient disinterest, personal disinterest

© 2024 Nguyen OT et al. *JAMA Network Open.*

Tamblyn et al., 2012	Canada	Physicians	Overprescribing of psychotropic drugs	unclear	Cluster RCT	81 physicians and 5628 patients total; intervention: 41 physicians and 2887 patients; control: 40 physicians and 2741 patients	Decrease behavior frequency	Yes	No	No	No	No	No	No	No	Yes	MOXXI	Yes	No	No	Not assessed	Not assessed
Sequist et al., 2012	United States	Physicians, PAs, NPs	Chest pain	2-month follow-up	Parallel RCT	292 total clinicians: 1) intervention group (149 clinicians; 3634 patients); 2) control group (143 clinicians, 3449 patients)	Increase behavior frequency	Yes	No	No	No	No	No	Yes	No	Yes	Epic	Yes	No	No	Not assessed	Not assessed

© 2024 Nguyen OT et al. *JAMA Network Open.*

Wright et al., 2012	United States	Not reported	ADHD, asthma/COPD, breast cancer, CAD, congenital coagulopathy, CHF, diabetes mellitus, glaucoma, hypertension, hyperthyroidism, hypothyroidism, myasthenia gravis, osteoporosis/ osteopenia, rheumatoid arthritis, renal failure/insufficiency, sickle cell disease, stroke	6 months	Cluster RCT	14 clinics and 38,025 patients in intervention arm, 14 clinics and 41,039 patients in control arm	Increase behavior frequency	No	No	No	No	No	Yes	No	No	Yes	LMR	Yes	No	No	Not assessed	Not assessed
Høye et al., 2013	Norway	Not reported	Upper respiratory infections	One year	Cluster RCT	40 groups of clinicians in intervention arm, 41 groups in control arm. Togethe	Decrease behavior frequency	Yes	No	No	No	No	No	Yes	No	No	Not reported	Yes	No	No	Not assessed	Not assessed

© 2024 Nguyen OT et al. *JAMA Network Open.*

						r, there were 328 GPs<br><br>156 GPs in intervention groups<br>172 GPs in Control																
Fiks et al., 2013	United States	Physicians	Human papillomavirus immunizations	12 month follow-up	Cluster RCT	22 practices total: 1) clinician focused intervention only (4495 families); 2) family focused intervention only (5714 families); 3) clinician and family focused intervention (5593 families)	Increase behavior frequency	Yes	No	No	Yes	No	No	No	No	Yes	Epic	Yes	No	No	Not assessed	Not assessed

© 2024 Nguyen OT et al. *JAMA Network Open.*

						; 4) control condition (5720 families)															
Hsu et al., 2013	United States	Physicians	Hepatitis B screening	3 months	Parallel RCT	38 clinicians and 87 patients in control arm, 38 clinicians and 88 patients in intervention arm	Increase behavior frequency	No	No	No	No	No	No	No	No	Yes	Epic	Yes	No	No	Not assessed	Not assessed
Zera et al., 2015	United States	Physicians	Type II diabetes screening	Not Applicable	Cluster RCT	26 practices total: Reminder condition (N=13 practices; 784 patients); Control Condition (N=13 practices; 973 patients)	Increase behavior frequency	No	No	No	No	No	No	No	No	Yes	Longitudinal Medical Record (LMR)	Yes	No	No	Not assessed	Not assessed

© 2024 Nguyen OT et al. *JAMA Network Open.*

Stockwell et al., 2015	United States	Physician, NPs, nurses	Influenza immunizations	1 year	Crossover RCT	6593 patients	Increase behavior frequency	Yes	No	No	No	No	No	Yes	No	Yes	Allscript Sunrise	Yes	No	No	Not assessed	Not assessed
Szilagyi et al., 2015	United States	Physicians, nurses	Pediatric immunizations	1 year	Cluster RCT	11 clinics in control arm, 11 clinics in intervention arm	Increase behavior frequency	Yes	No	No	No	No	No	No	No	Yes	Not reported	Yes	No	No	Not reported	Lack of time, errors in how prompts are fired, lack of complete intervention acceptance by clinicians and staff
Meeker et al., 2016	United States	Physicians	Upper respiratory infections	18 month follow-up	Cluster RCT	Total: 49 practices: 6 practices; 27 clinicians (control condition); 6 practices; 42 clinicians (suggested alternati	Decrease behavior frequency	No	No	No	Yes	No	No	No	Yes	Yes	Mixed	No	No	Yes	Not assessed	Not assessed

© 2024 Nguyen OT et al. *JAMA Network Open.*

						ves condition); 7 practices; 35 clinicians (accountable justification condition); 4 practices; 24 clinicians (peer comparison condition)														
Guiriguet et al., 2016	Spain	Physicians, nurses	Colorectal cancer screening	1 year	Parallel RCT	67 GPs and 21,619 patients in intervention arm, 63 GPs and 19,423 patients in control arm	Increase behavior frequency	No	No	No	No	No	No	No	No	Yes	Not reported	Yes	No	No	Not assessed	Not assessed

© 2024 Nguyen OT et al. *JAMA Network Open.*

Kressin et al., 2016	United States	armac	Hypertension	1 year	Cluster RCT	8866 patients, sample size per group not reported	Increase behavior frequency	Yes	No	No	No	No	No	Yes	Yes	Yes	Not reported	Yes	No	No	Not assessed	Not assessed
Federman et al., 2017	United States	Physicians, medical assistants	Hepatitis C Virus testing	11 months	Cluster RCT	82 clinicians were in intervention arm and 45 clinicians were in control arm. There were also 14,825 patients in intervention arm and 10,795 patients in control arm	Increase behavior frequency	Yes	No	No	No	Yes	No	Yes	Yes	Yes	Epic	Yes	No	Yes	Not assessed	Not assessed
Kharbanda	United States	Physicians, nurses	Hypertension	90-days	Cluster RCT	20 practices total: intervent	Increase behavior	Yes	No	No	No	No	No	No	No	Yes	TeenBP	Yes	No	Yes	Not assessed	Not assessed

© 2024 Nguyen OT et al. *JAMA Network Open.*

et al., 2018						ion: 607 patients, control: 607 patients	frequency															
Karlsson et al., 2018	Sweden	Physicians	Atrial fibrillation	1 year	Cluster RCT	22 clinics in intervention, 21 clinics in usual care	Increase behavior frequency	Yes	Yes	No	No	No	No	Yes	Yes	Yes	Cambio Health care Systems	Yes	No	No	Not assessed	Not assessed
Sequist et al., 2018	United States	Physicians	Chronic kidney disease	1 year	Parallel RCT	76 physicians and 3,913 patients in intervention arm, 77 physicians and 3,778 patients in control arm	Increase behavior frequency	No	No	No	No	Yes	No	No	No	Yes	Epic	Yes	No	No	Not assessed	Not assessed
Chak et al., 2018	United States	Not reported	Hepatitis B screening	1 year	Parallel RCT	1,484 patients in intervention arm, 1,503 randomized in	Increase behavior frequency	No	No	No	No	No	No	No	No	Yes	Epic	Yes	No	No	Not assessed	Not assessed

© 2024 Nguyen OT et al. *JAMA Network Open.*

						control arm																
Tamblyn et al., 2018	Canada	Physicians	Uncomplicated hypertension	60 months	Parallel RCT	38 physicians and 1,914 patients in intervention arm, 38 physicians and 1,678 patients in control arm	Increase behavior frequency	Yes	No	No	Yes	No	Yes	Yes	Yes	Yes	Not reported	Yes	No	Yes	Not reported	Having only one option to choose from, which prevents clinical judgment from being used to determine best option for patients
Gulliford et al., 2019	United Kingdom	Not reported	Upper respiratory infections	Not applicable	Cluster RCT	41 intervention clinics, 38 control clinics<br><br>Unknown number of clinicians in each arm	Decrease behavior frequency	Yes	No	No	Yes	No	No	No	No	Yes	Not reported	Yes	No	No	Not assessed	Not assessed

© 2024 Nguyen OT et al. *JAMA Network Open.*

Zafar et al., 2019	United States	Physicians	Lower back pain	The intervention period varied for each condition: Baseline- 8 months Intervention period 1 with report cards for group 1 and CDS alerts for group 2- 10 months Intervention period 2- 12 months both groups recieve	Crossover RCT	8 PCP practices with average of 142.000 annual adult outpatient visits.	Decrease behavior frequency	No	No	No	Yes	No	No	No	No	Yes	Not reported	Yes	No	No	Not assessed	Not assessed

© 2024 Nguyen OT et al. *JAMA Network Open.*

				reports cards and CDS alerts																		
Weiner et al., 2020	United States	Physicians	Hypoglycemia	Five-month follow-up assessment	Parallel RCT	343 total clinicians: 1) intervention condition (123 clinicians, 3350 patients); and 2) control condition (220 clinicians, 3395 patients)	Increase behavior frequency	No	No	No	No	No	No	No	No	Yes	Regenstrief G3 system	Yes	No	No	Not assessed	Not assessed
McKie et al., 2020	United States	Physicians, APPs	Cardiovascular disease	7-day follow-up after patient visit	Cluster RCT	20 care teams: 1) intervention condition (10 care teams; 55 clinicians); 2) control conditio	Increase behavior frequency	Yes	No	No	No	No	No	No	No	Yes	General Electric Centricity	Yes	No	No	Not assessed	Not assessed

© 2024 Nguyen OT et al. *JAMA Network Open.*

						n (10 care teams; 54 clinicians)																
Chak et al., 2020	United States	Not reported	Hepatitis B screening	3 years	Parallel RCT	Cohort 1: 1,542 intervention patients, 1,568 control patients Cohort 2: 2,599 intervention patients, 2,590 control patients	Increase behavior frequency	No	No	No	No	No	No	No	No	Yes	Epic	Yes	No	No	Not assessed	Not assessed
Stephens et al., 2021	United States	Physicians, NPs	Pediatric immunizations	1 year	Crossover RCT	Not reported	Increase behavior frequency	No	No	No	No	No	No	No	No	Yes	Not reported	Yes	No	No	Not reported	Concerns on accuracy of reminder
Campbell et al., 2021	United States	Not reported	Anticholinergic prescribing	12 months	Cluster RCT	254 patients in intervention arm, 298 patients in	Decrease behavior frequency	No	No	No	No	Yes	Yes	No	No	Yes	Not reported	Yes	No	Yes	Not assessed	Not assessed

© 2024 Nguyen OT et al. *JAMA Network Open.*

						control arm																
Dhingra et al., 2021	United States	Physicians, NPs, PAs	Musculoskeletal or neuropathic pain	6 months	Crossover RCT	Early Implementation: n=256 patients and 3 practices Delayed Implementation: n=272 patients and 3 practices	Increase behavior frequency	Yes	Yes	No	No	No	No	No	No	Yes	Epic	Yes	Yes	Yes	Not assessed	Not assessed
Kraemer et al., 2022	United States	Not reported	Musculoskeletal pain, headache	12 months	Cluster RCT	12 practices and 5,082 patients in control arm, 12 practices and 7,006 patients in arm with opioid justification, 12 practices and	Decrease behavior frequency	No	No	No	Yes	No	No	Yes	Yes	Yes	Not reported	Yes	No	Yes	Not assessed	Not assessed

© 2024 Nguyen OT et al. *JAMA Network Open.*

						5,704 patients in arm with clinician comparison, and 12 practices and 4,828 patients in arm with opioid justification and clinician comparison														
Weiner et al., 2022	United States	Physicians	Not reported	6 months	Parallel RCT	177 patients in intervention arm, 275 patients in control arm	Increase behavior frequency	No	No	No	No	No	No	No	No	Yes	Cerner, Epic	Yes	No	No	Not assessed	Not assessed
Lee et al., 2023	United States	Not reported	Alcohol screening	2 yrs, 7 months	Crossover RCT	19 sites and 255,789 patients in usual care arm, the	Increase behavior frequency	Yes	No	No	Yes	No	No	No	No	Yes	Not reported	Yes	No	No	Not assessed	Not assessed

© 2024 Nguyen OT et al. *JAMA Network Open.*

						same 19 sites and 228,258 patients in intervention arm																
Sigel et al., 2023	United States	Not reported	Firearm screening	3 years	Parallel RCT	15 visits in control arm, 41 in intervention arm	Increase behavior frequency	Yes	No	No	No	No	No	No	No	Yes	Not reported	Yes	Yes	No	Not assessed	Not assessed
Adusumalli et al., 2023	United States	Physicians	Statin prescribing	6 months	Cluster RCT	7 clinics, 39 clinicians, and 1,032 patients in control arm<br><br>7 clinics, 44 clinicians, 1,181 patients in patient nudge arm<br><br>7 clinics, 32	Increase behavior frequency	No	No	No	Yes	No	No	No	No	Yes	Epic	Yes	No	No	Not assessed	Not assessed

© 2024 Nguyen OT et al. *JAMA Network Open.*

clinician
s, 981
patients
in
clinician
nudge
arm

7 clinics,
41
clinician
s, 937
patients
in
patient
and
clinician
nudge
arm

© 2024 Nguyen OT et al. *JAMA Network Open.*

eTable 3. Study quality assessment results

Study	Randomization Process	Timing of Identification or Recruitment of Participants	Carryover Effects	Assignment to Interventions	Adhering to Interventions	Missing Outcome Data	Measurement of Outcome	Selection of Reported Result	Overall Risk of Bias
Adusumalli et al., 2023	Low	Low	N/A	Low	Low	Low	Low	Low	Low
Bell et al., 2010	Some	Low	N/A	Low	Some	Low	Low	Some	Some
Campbell et al., 2021	Some	Low	N/A	Low	Some	Low	Low	Some	Some
Co et al., 2010	Low	Low	N/A	Low	Some	Low	Low	Some	Some
Feder et al., 2011	Low	Low	N/A	Low	Some	Low	Low	Low	Some
Federman et al., 2017	Some	Low	N/A	Low	Some	Low	Low	Some	Some
Fiks et al., 2009	Low	Low	N/A	Low	Some	Low	Low	Some	Some
Fiks et al., 2013	Low	Low	N/A	Low	Some	Low	Low	Some	Some
Flottorp et al., 2002	Low	Low	N/A	Low	Some	High	Low	Some	High
Fortuna et al., 2009	Some	Low	N/A	Some	High	Low	Low	Low	High
Gill et al., 2009	Some	Low	N/A	Low	Some	Low	Low	Some	Some
Gill et al., 2011	Low	Low	N/A	Low	Some	Low	Low	Some	Some
Gulliford et al., 2019	Low	Low	N/A	Low	Some	Low	Low	Some	Some
Hoye et al., 2013	Low	Low	N/A	Low	Some	Low	Low	Some	Some
Karlsson et al., 2018	Low	Low	N/A	High	High	Low	Low	Some	High
Kharbanda et al., 2018	Some	Low	N/A	Low	Some	Low	Low	Some	Some
Kraemer et al., 2022	Low	Low	N/A	Low	Some	Low	Low	Some	Some
Kressin et al., 2016	Some	Low	N/A	Low	Some	Low	Low	Some	Some
Linder et al., 2009	Low	Low	N/A	Low	Some	Low	Low	Some	Some
Lo et al., 2009	Some	Low	N/A	Low	Some	Low	Low	Some	Some
McKie et al., 2020	Some	Low	N/A	Low	Some	Low	Low	Some	Some
Meeker et al., 2016	Low	Low	N/A	Low	Some	Low	Low	Some	Some
O'Connor et al., 2011	Some	Low	N/A	Low	Some	High	Low	Some	High
Player et al., 2010	Low	Low	N/A	Low	Some	Low	Low	Some	Some
Schriefer et al., 2009	Low	Low	N/A	Low	Some	Low	Low	Some	Some
Sequist et al., 2005	Some	Low	N/A	Low	Some	Low	Low	Some	Some

© 2024 Nguyen OT et al. *JAMA Network Open.*

Szilagyi et al., 2015	Some	Low	N/A	Low	Some	Low	Low	Some	Some
Tamblyn et al., 2012	Low	Low	N/A	Low	Some	Low	Low	Some	Some
Tamblyn et al., 2003	Low	Low	N/A	Some	High	Low	Low	Low	High
Tang et al., 2012	Low	Low	N/A	Low	Some	Low	Low	Some	Some
van Wyk et al., 2008	Some	Low	N/A	Low	Some	Low	Low	Some	Some
Wright et al., 2012	Low	Low	N/A	Low	Some	Low	Low	Some	Some
Zera et al., 2015	Some	Low	N/A	Low	Some	High	Low	Some	High
Palen 2006	Low	N/A	N/A	High	High	Low	Low	Some	High
Sequist 2012	Low	N/A	N/A	Low	Some	Low	Low	Some	Some
Hsu 2013	Low	N/A	N/A	Low	Some	Low	Low	Some	Some
Frank 2004	Some	N/A	N/A	Low	Some	Low	Low	Some	Some
Weiner 2020	Low	N/A	N/A	Low	Some	Low	Low	Some	Some
Sequist 2018	Low	N/A	N/A	Low	Some	Low	Low	Some	Some
Chak 2018	Low	N/A	N/A	Low	Some	Low	Low	Some	Some
Tamblyn 2018	Low	N/A	N/A	Low	Some	Low	Low	Some	Some
Guiriguet 2016	Low	N/A	N/A	Low	Some	Low	Low	Some	Some
McDowell 1989	Some	N/A	N/A	Low	Some	Low	Low	Some	Some
Chak 2020	Low	N/A	N/A	Low	Some	Low	Low	Some	Some
Abdel-Kader 2011	Some	N/A	N/A	Low	Some	Low	Low	Some	Some
Loo 2011	Low	N/A	N/A	Low	Some	Low	Low	Some	Some
Sigel 2023	Low	N/A	N/A	Low	Some	Low	Low	Some	Some
Weiner 2022	Low	N/A	N/A	Low	Some	Low	Low	Low	Some
Feldstein et al., 2006	Low	N/A	N/A	Low	Some	Low	Low	Some	Some
Zafar 2019	Low	N/A	Low	Low	Some	High	Low	Some	High
Stockwell 2015	Low	N/A	High	Low	Some	Low	Low	Some	High
Dhingra 2021	Some	N/A	Low	Low	Some	High	Low	Some	High
Stephens 2021	Low	N/A	High	Low	Some	Low	Low	Some	High
Lee 2023	Low	N/A	Low	Low	Some	Low	Low	Low	Some

© 2024 Nguyen OT et al. *JAMA Network Open.*
