# Supplementary material for: Electronic Health Record Nudges and Health Care Quality and Outcomes in Primary Care: A Systematic Review
Source: JAMA Netw Open. 2024 Sep 17;7(9):e2432760. doi: 10.1001/jamanetworkopen.2024.32760 (PMC11409160; doi:10.1001/jamanetworkopen.2024.32760)
Supplement: Supplement 2. — Data Sharing Statement [file jamanetwopen-e2432760-s002.pdf]

Data Sharing Statement

Nguyen. Electronic Health Record Nudges and Health Care Quality and Outcomes in Primary Care. *JAMA Netw Open*. Published September 11, 2024.
doi:10.1001/jamanetworkopen.2024.32760

Data
Data available: No
